# Spontaneous Reduction of Cu(II) Complexes with Imidazole-Derived Ligands in Acetonitrile

**DOI:** 10.3390/molecules31081245

**Published:** 2026-04-09

**Authors:** Brenda Sánchez-Eguía, Carolina Sánchez-López, Marcos Flores-Álamo, Nils Schuth, Víctor M. Ugalde-Saldívar, Virginia Gómez-Vidales, Chiara E. Campi, Juan Raúl Álvarez Idaboy, Liliana Quintanar, Laura Gasque

**Affiliations:** 1 Facultad de Química, Universidad Nacional Autónoma de México, Ciudad de Mexico 04510, Mexico; mfa@unam.mx (M.F.-Á.); vmus@unam.mx (V.M.U.-S.); jidaboy@unam.mx (J.R.Á.I.); 2 Center for Research in Aging, Ciudad de Mexico 14330, Mexico; mcsanchez@cinvestav.mx (C.S.-L.); lilianaq@cinvestav.mx (L.Q.); 3 Department of Chemistry, Center for Research and Advanced Studies (Cinvestav), Ciudad de Mexico 07350, Mexico; nils.schuth@gmail.com; 4 Instituto de Química, Universidad Nacional Autónoma de México, Circuito Exterior, Ciudad Universitaria, Ciudad de Mexico 04510, Mexico; gomezvv@unam.mx; 5 Institute of Inorganic and Analytical Chemistry, Justus Liebig University Giessen, 35390 Giessen, Germany; chiara.campi@anorg.chemie.uni-giessen.de

**Keywords:** copper, imidazole, electron transfer, spontaneous reduction, acetonitrile, superoxide

## Abstract

The spontaneous reduction of one Cu(II) center to Cu(I) in a series of three dinuclear copper complexes in acetonitrile is described. These complexes feature ligands that include nitrogen donors from a diazecine ring and imidazole, designated as *promeim*, *thiopromeim*, and *thioenmeim*; the latter two incorporate a thioether as a third donor component. The mechanism of metal reduction was elucidated through spectroscopic and spectrometric techniques (UV-vis, EPR, XANES, ESI-MS) and electrochemical tools, in combination with DFT electronic structure calculations. Based on these and on spectroelectrochemical results, a mechanism is proposed in which the one-electron reduction of one of the copper ions is achieved by a one-electron oxidation in the adjacent imidazole group, while the other copper ion remains as Cu(II). The persistent detection of superoxide and peroxide over long periods suggests a mechanism in which a catalytic cycle involving electron transfer occurs between copper, ligand, and dioxygen.

## 1. Introduction

The main function of copper enzymes in biological systems is that of electron transfer, due to this metal ion’s ability to reversibly change oxidation states from II to I. It is noteworthy that in all of these enzymes, copper is always bound to at least one imidazole group from histidine residues [1,2,3,4].

Among the abundant copper complexes described in the literature over the years, there have been quite a few reports of a puzzling redox phenomenon that has been described as “autoreduction” or “spontaneous reduction” of Cu(II) to Cu(I) in a wide variety of chemical environments. Many of these involve bioinspired coordination complexes [5,6,7,8,9,10,11,12,13,14,15,16,17,18,19,20,21], and in some cases, the redox-active character of the ligands promotes metal reduction [22,23]. Even though in some of these articles the role of the solvent is mentioned and structural arguments are given for the stabilization of Cu(I) over Cu(II), most of these reports have not made clear the mechanism of electron transfer, nor do they identify the reducing species responsible for this transformation. In limited examples, electron transfer from the ligand to Cu(II) to create a free radical intermediate has been proposed [24,25,26]. The related phenomenon of coordination compound redox isomerism or valence tautomerism, which involves the interconversion of two electronic isomers or valence tautomers (i.e., species with identical chemical composition) with different charge distributions, has been amply described since the 1980s [27] and continues to be relevant [28,29]. Also recently, autoreduction of copper (II) has been reported on zeolite or molecular sieve-adsorbed copper salts, where coordinated -OH groups from the zeolite act as electron donors while being oxidized to molecular oxygen [30,31,32,33,34].

**Scheme 1:**
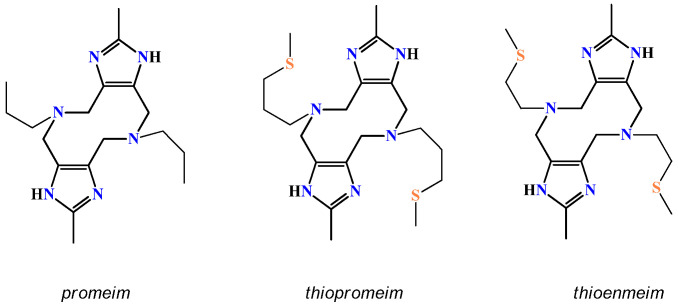
Dinucleating ligands used in this work.

In this work, we describe the “autoreduction” reaction experienced by three dinuclear Cu(II) complexes with imidazole-containing ligands, *promeim*, *thiopromeim*, and *thioenmeim* (Scheme 1), when formed in acetonitrile using non-coordinating counteranions. Freshly prepared solutions exhibit a deep green color common for Cu(II) complexes and turn blue over time, following the kinetic pattern Cu_2_*promeim* >> Cu_2_*thiopromeim* >> Cu_2_*thioenmeim.* Attempts to isolate the product by slow evaporation of the solvent yielded a blue slurry and colorless crystals containing [Cu(MeCN)_4_]^+^ (Figure S1).

Analysis of this behavior was performed by spectroscopic, electrochemical, and computational means. These results, strongly supported by spectroelectrochemical experiments, led to a proposed mechanism in which the reduction of Cu(II) is accomplished by the loss of an electron from the ligand, possibly generating an intermediate species involving a radical cation in the imidazole moiety. In aerobic solutions, Cu(I) generated in this way reduces atmospheric O_2_, producing the superoxide ion. The latter can be detected by EPR spin trapping with DMPO at virtually any given moment, suggesting Cu(I) persists for long periods despite its reaction with dioxygen, and thus must be regenerated in a redox cycle involving Cu(II)/Cu(I), Imz/Imz^·^ and O_2_/O_2_^·−^. This work addresses a transformation that takes place specifically in the presence of acetonitrile, favored by the well-known stabilizing effect this coordinated solvent has on Cu(I). These findings may be relevant to copper enzyme chemistry, since the imidazole group from histidine is a ubiquitous coordinating group in Cu redox enzymes. Even when acetonitrile is never present in these systems, there are steric and electronic factors that promote the necessary metal coordination geometry changes that enable reversible changes in oxidation state.

## 2. Results

### 2.1. Characterization of the Complexes in Their Initial State

#### 2.1.1. Crystal Structures

**Figure 1:**
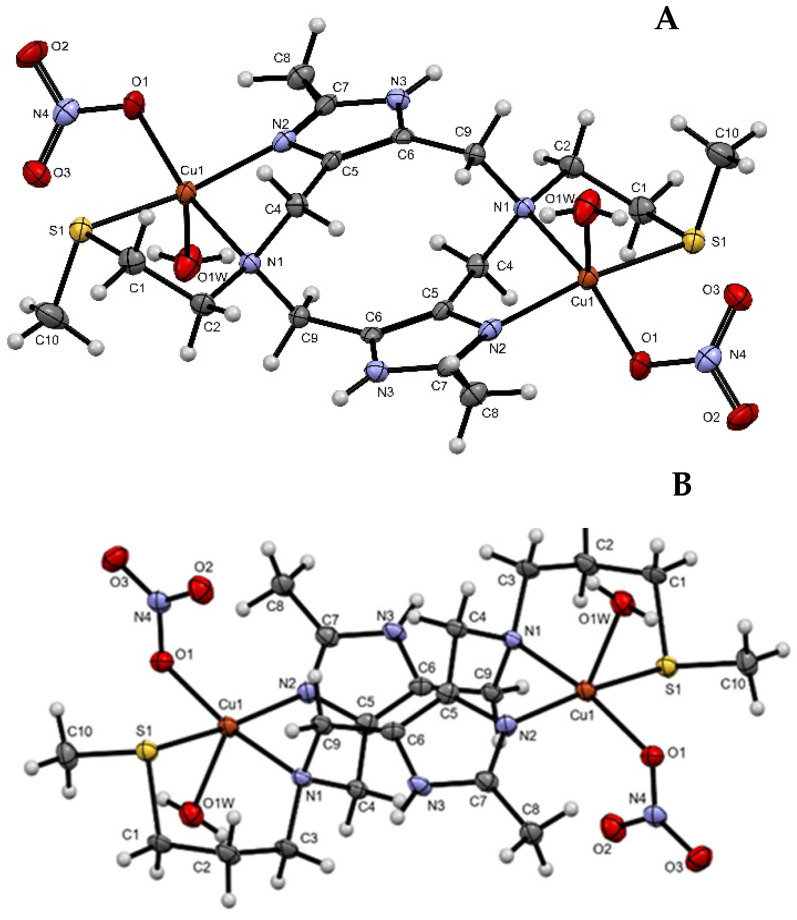
Perspective views for the structures of [Cu_2_*thioenmeim*(H_2_O)_2_(NO_3_)_2_](NO_3_)_2_ (**A**) and [*Cu*_2_*thiopromeim*(H_2_O)_2_(NO_3_)_2_](NO_3_)_2_ (**B**), with displacement ellipsoids at the 70% probability level for non-H atoms.

Crystals of the thioether-containing ligand complexes [Cu_2_(*thioenmeim*)(H_2_O)_2_(NO_3_)_2_](NO_3_)_2_ and [Cu_2_(*thiopromeim*)(H_2_O)_2_(NO_3_)_2_](NO_3_)_2_ were easily obtained from methanol–water solutions. X-ray analysis revealed similar structures with the 10-membered central diazecine ring adopting a chair conformation, consistent with several complexes obtained by our group [35,36,37,38,39,40] (Figure 1A,B, CCDC: 2442956–2442957). Both complexes form centro-symmetric dinuclear molecules in which copper ions are five-coordinate in slightly distorted square-pyramidal geometry with τ parameter values [5] of 0.177 and 0.031, respectively. The Cu...Cu distances are 7.5129(6) Å for the former and 7.313(2) Å for the latter. In both complexes, the base of each pyramid is formed by two nitrogen atoms from the ligand, including a N(2) atom from the imidazole group and a N(1) atom from the tertiary amine, as well as an oxygen atom -O(1) from the nitrate group and a sulfur atom from the thiomethyl group. The apex of the pyramid is occupied by an oxygen atom from a coordinated water molecule, O(1W). Relevant bond distances and angles are listed in Table S2. Crystal packing and the description of network intermolecular interactions for these two complexes are presented in the Supplementary Materials (Figure S2A,B).

Crystals of the analogous copper complex with the *promeim* ligand proved elusive, probably due to this ligand’s lack of a third donor.

#### 2.1.2. Electronic Spectra

**Figure 2:**
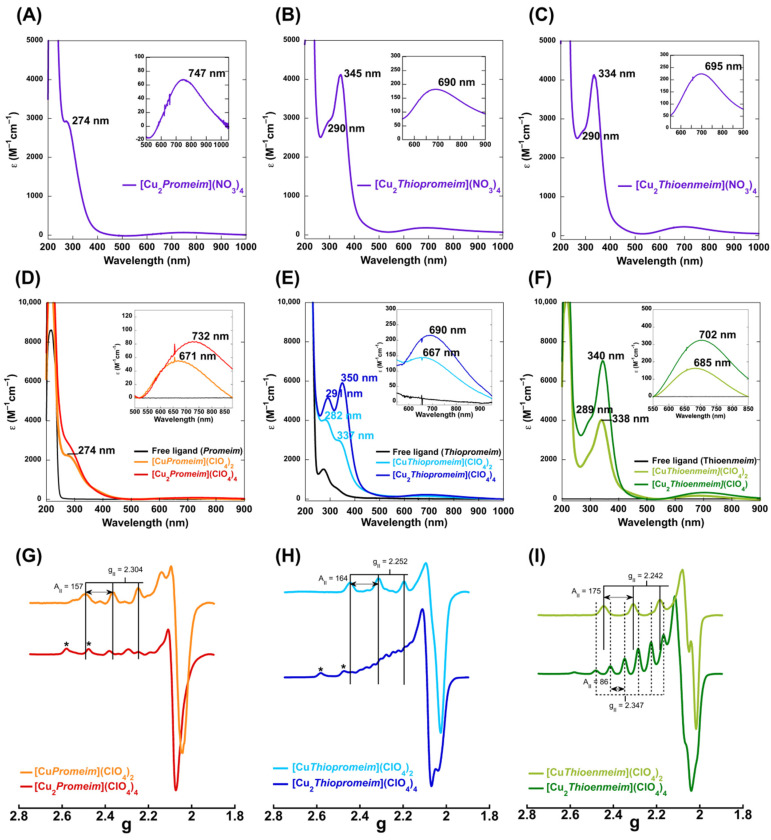
Initial characterization of Cu^2+^-ligand complexes by UV-vis absorption and EPR spectroscopy. UV−Vis absorption spectra of methanolic solutions of Cu(II) complexes with *promeim* (**A**), *thiopromeim* (**B**), and *thioenmeim* (**C**), with 2.0 equiv. of Cu(NO_3_)_2_ salt. Corresponding UV-vis of solutions with 1.0 and 2.0 equivalents of Cu(ClO_4_)_2_ salt are shown in (**D**–**F**). (**G**–**I**) show the 150 K spectra of copper:ligand solutions in 1:1 and in 2:1 ratios for *promeim*, *thiopromeim*, and *thioenmeim*, respectively. Solutions in (**D**–**I**) were prepared with Cu(ClO_4_)_2_. A_II_ values in EPR spectra are given in 1 × 10^−4^ cm^−1^. Asterisks in (**G**,**H**) indicate signals associated with copper in solution.

UV-vis electronic absorption spectra were initially obtained under the same conditions as those of crystallization, namely in methanol solution and with nitrates as counterions. The absorption spectrum of the [Cu_2_*promeim*](NO_3_)_4_ complex (Figure 2A) displays a ligand field absorption at 747 nm (ε = 67 M^−1^ cm^−1^) and a ligand-to-metal charge transfer (LMCT) band at 274 nm (ε = 2915 M^−1^ cm^−1^) assigned to an imidazole π to Cu(II) [41].

For the [Cu_2_*thiopromeim*](NO_3_)_4_ and [Cu_2_*thioenmeim*](NO_3_)_4_ complexes (Figure 2B,C), the ligand field transitions are shifted to 690 nm (ε = 180 M^−1^ cm^−1^) and to 695 nm (ε = 220 M^−1^ cm^−1^), respectively, while LMCT bands are observed at 345 nm (ε = 4118 M^−1^ cm^−1^) for [Cu_2_*thiopromeim*](NO_3_)_4_ and 334 nm (ε = 4132 M^−1^ cm^−1^) for [Cu_2_*thioenmeim*](NO_3_)_4_, corresponding to S → Cu(II) LMCT transitions [42]. Additionally, in both spectra, one shoulder is observed at 290 nm, corresponding to the imidazole π to Cu(II) LMCT transition.

A comparison of the electronic absorption spectra of solutions with 1:1 and 2:1 Cu:ligand ratios for the three ligands, as prepared in ethanol and using Cu(ClO_4_)_2_ as the copper source, is shown in Figure 2D–F. The *promeim* complexes at 1:1 and 2:1 Cu:ligand ratios exhibit very similar spectroscopic features, showing a slight increase in absorbance at 274 nm, assigned to an imidazole π-to-Cu(II) LMCT. Meanwhile, the ligand-field transitions shift from 671 to 732 nm. For the *thiopromiem* and *thioenmeim* complexes, larger differences are observed in the LMCT region. Specifically, the LMCT associated with the sulfur-to-Cu(II) transition increases in intensity and shifts slightly to a higher wavelength, from 337 to 350 nm for the *thiopromeim* complexes (Figure 2E) and from 338 to 340 nm for the *thioenmeim* complexes (Figure 2F). A similar trend (i.e., a shift to lower energy/higher wavelength is observed for the ligand field transitions when going from the 1:1 and 2:1 Cu:ligand ratios.

#### 2.1.3. EPR Spectra

Solutions with 1:1 and 2:1 Cu:L ratios were also studied by electron paramagnetic resonance (EPR), using ethanol as a solvent and perchlorates as counterions. Under these conditions, their EPR spectra display signals with g_||_ > g_⊥_ > 2.0023 and a large parallel hyperfine splitting (A_||_), indicative of tetragonal Cu(II) centers with a d_x2−y2_ ground state [18]. The EPR spectrum of the Cu:*promeim* solution in a 1:1 ratio displays A_||_ = 157 × 10^−4^ cm^−1^ and g_||_ = 2.304 values, corresponding to a nitrogen-rich coordination mode. In contrast, the EPR spectrum of the Cu:*thiopromeim* 1:1 displays different A_||_ and g_||_ values of 164 × 10^−4^ cm^−1^ and 2.252, respectively, possibly reflecting an equatorial coordination sphere that includes nitrogen- and sulfur-based ligands. In addition, the other ligand that contains sulfur atoms in its structure, Cu:*thioenmeim* 1:1, displays signals with parameters very close to Cu:*thiopromeim*, with A_||_ = 175 × 10^−4^ cm^−1^ and a g_||_ = 2.242, supporting the participation of sulfur atoms in the coordination sphere to Cu(II) in a 1:1 ligand:metal ratio.

In contrast, the 2:1 Cu:ligand solutions show strikingly different EPR features, as compared to those of the 1:1 samples. In particular, for the *thioenmeim* ligand, a clear seven-line signal is observed in the parallel region of the spectra, indicative of electron spin coupling to two nuclear Cu(II) spins (I = 3/2). Several similar EPR spectra have been previously described by our group for dinuclear copper(II) complexes with analogous ligands [35,36,37,38,39,40] and by other research groups for other complexes with aliphatic spacers [43,44,45], in which separation distances range from 6.9 to 9.1 Å. The EPR spectrum of the 2:1 Cu:*thioenmeim* solution (green spectrum in Figure 2I) can be simulated by considering two Cu(II) centers, with their electron spins coupled to both metal nuclear spins, exhibiting a hyperfine splitting of A_||_ = 86 × 10^−4^ cm^−1^ and a g_||_ = 2.347 (Figure S3, Table S3). It should be noted that this hyperfine pattern is not evident in the spectra of the 2:1 Cu:*promeim* and Cu:*thiopromeim* solutions but could be observed only when acetates were used as counterions (Figure S4). In these two complexes, where there is no third donor atom (Cu_2_*promeim*) or where the third donor atom forms a larger, and thus less stable, chelate ring (Cu_2_*thiopromeim*), greater internal mobility might cause different conformations in which magnetic coupling cannot take place.

There may also have been a small amount of mononuclear species and a small amount of free copper in the solution (Figure 2G,H).

### 2.2. Transformations in MeCN

#### 2.2.1. Electronic Spectra

**Figure 3:**
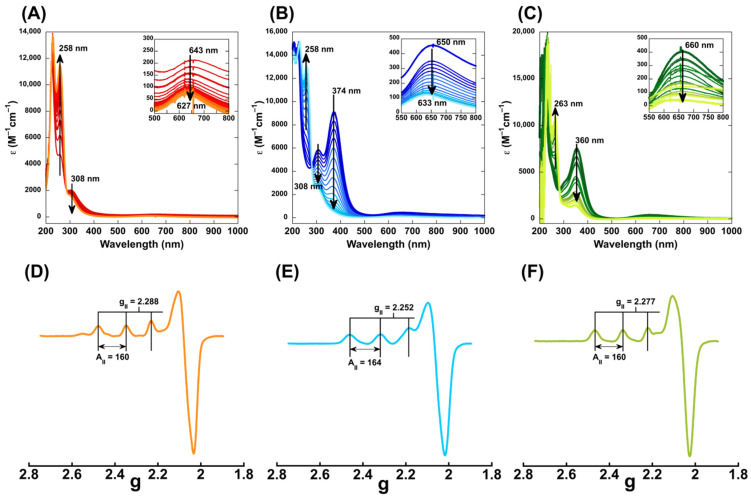
Transformation of the dinuclear complexes, monitored by UV-vis absorption and electron paramagnetic resonance (EPR). UV-vis absorption spectra showing the transformation of [Cu_2_*promeim*] (**A**), [Cu_2_*thiopromeim*] (**B**), and [Cu_2_*thioenmeim*] (**C**) complexes in MeCN/EtOH (80%/20%) over time. The EPR spectra at 150 K were recorded at the end of the reaction: (**D**–**F**). A_II_ values are given in 1 × 10^−4^ cm^−1^.

To gain insight into the reactivity of the dinuclear complexes under different conditions, their behavior in MeCN solvent was investigated. The dinuclear Cu complexes with *promeim*, *thiopromeim*, and *thioenmeim*, as described above, are stable in methanol or ethanol and readily obtained upon mixing the ligand with two equivalents of Cu(II) with non-coordinating anions (i.e., ClO_4_^−^, BF_4_^−^ or OTf^−^). *Caution!* Perchlorates are explosive; handle with care. However, when prepared in acetonitrile, their solutions undergo marked color and spectroscopic changes, going from green to blue. This transformation was followed by UV-vis electronic absorption spectroscopy in the presence of 80% acetonitrile and 20% ethanol (since the ligand was insoluble in MeCN but soluble in EtOH), revealing the relative rate at which the three complexes undergo this change: Cu_2_*promeim* (reaction time ca. ½ h) << Cu_2_*thiopromeim* (ca. 3 h) << Cu_2_*thioenmeim* (>>24 h). Figure 3A–C show the changes in the UV-vis spectra of the MeCN/EtOH solutions of the three complexes. In the UV, the most noticeable changes for Cu_2_*thiopromeim* and Cu_2_*thioenmeim* are marked decreases in the 370 nm band associated with S → Cu LMCT, which is indicative of the loss of the thioether group from the coordination shell. For the three complexes, a decrease in 308 nm absorption, associated with imidazole → Cu LMCT, is observed, while there is a concomitant emergence of an intense band at ca. 258 nm.

In the visible region, the *d-d* band decreases in intensity, suggesting a partial reduction of Cu(II) to Cu(I), and its blue shift is indicative of a change in the coordination environment for the remaining Cu(II) species. The longer reaction rates observed for complexes with thioether-containing ligands may have been caused by a preliminary step where Cu-S bonds were substituted for Cu-NCMe bonds. This decoordination is expected to be slower for Cu_2_*thioenmeim* than for Cu_2_*thiopromeim* since, in the former, the sulfur atom forms part of a five-membered chelate ring with the metal ion, while in the latter, the chelate ring is six-membered. It is typical for complexes with analogous ligands that stability constants are larger for the complex with the five-membered ring-forming ligand. This trend has been observed in our lab in complexes with similar ligands [40]. After the loss of the thioether groups’ coordination, the three complexes can attain equivalent coordination environments.

It should be noted that at longer time points (ca. three weeks), when most of the solvent had slowly evaporated in open air, the acetonitrile solutions of these complexes yielded the colorless crystals of [Cu(MeCN)_4_]X (X = ClO_4_^−^ or BF_4_^−^) (Figure S1), further supporting that metal reduction occurs. From the remaining deep-blue semisolid, it was impossible to crystallize any species, so the new absorption band at 258 nm could potentially be associated with ligand oxidation (see below).

For the *promeim* complex, analysis by MS of this aged semisolid after extracting the copper ions showed that the ligand remained mostly intact, with less than 10% transformation (Figure S5).

#### 2.2.2. EPR Spectra

The reactivity of the dinuclear Cu(II) complexes in MeCN/EtOH (80%/20%) solution was also studied by EPR spectroscopy. At the end of the transformation, the EPR spectra at 150 K were measured (Figure 3D–F), showing a single Cu(II) species with g_||_ = 2.288 and A_||_ = 160 × 10^−4^ cm^−1^ for the *promeim* complex, g_||_ = 2.252 and A_||_ = 164 × 10^−4^ cm^−1^ for the *thiopromeim* complex, and g_||_ = 2.277 and A_||_ = 160 × 10^−4^ cm^−1^ for the *thioenmeim* complex. These spectroscopic features are very similar to those observed for the Cu:ligand solutions in a 1:1 ratio (Figure 2G–I), suggesting the presence of a single Cu(II) ion in the complex at the end of the transformation. This scenario is consistent with one of the copper ions reducing to Cu(I).

#### 2.2.3. X-Ray Absorption Spectroscopy (XAS)

**Figure 4:**
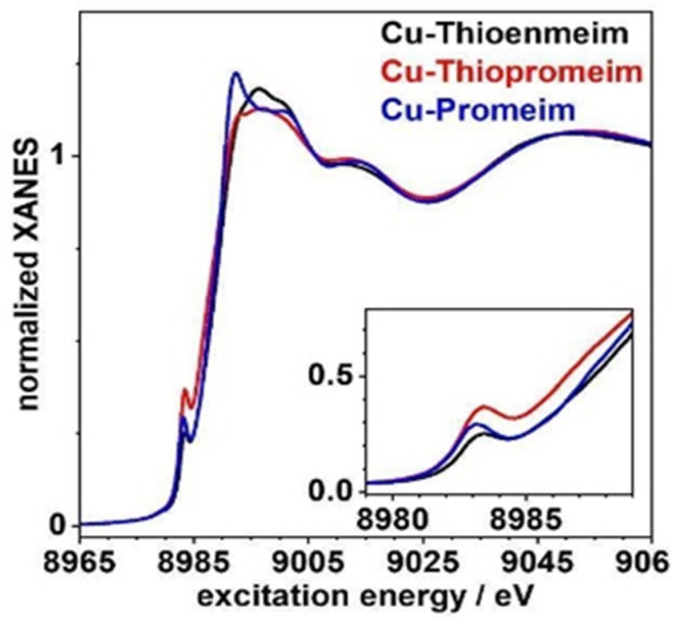
Normalized copper K-edge XANES spectra of the copper complexes with *promeim*, *thiopromeim*, and *thioenmeim* in acetonitrile, at a 2:1 Cu:ligand stoichiometry and 5 mM concentration, using Cu(ClO_4_)_2_ as a metal source, frozen three days after preparation.

Copper K-edge XAS was also used to corroborate the formation of Cu(I) species upon the reaction of the dinuclear complexes in MeCN. The complexes were prepared using Cu(ClO_4_)_2_ dissolved in acetonitrile and mixed with the corresponding ligand in a 2:1 Cu:ligand stoichiometry to yield the dinuclear complexes at a high concentration (5 mM). Samples were frozen for XAS analysis after three days of transformation in MeCN. The presence of Cu(I) ions at the end point of the reaction can be confirmed from X-ray absorption near-edge structure (XANES) spectra (Figure 4).

For all three complexes, a rising edge feature at about 8982–8984 eV is observed, which can be assigned to the 1 s → 4 p electronic transition of Cu(I) ions present in the samples [46]. Previous studies by Solomon and coworkers showed that rising edge peak energies and intensities can be reasonably correlated with the coordination number [47]. Based on this, the edge features observed in Figure 4 (inset) can be ascribed to a tricoordinated Cu(I) species. In contrast, for Cu(II) species, it is important to mention that the pre-edge feature associated with the 1 s → 3 d transition (~8979 eV) of Cu(II) species is hardly observed due to its low intensity, since it is an electric dipole forbidden transition. Overall, these XAS data confirm that, upon reaction in MeCN, these dinuclear Cu complexes undergo reduction of one of the Cu(II) centers to Cu(I). Such metal reduction may occur at the expense of ligand oxidation, as will be proposed next.

#### 2.2.4. Mass Spectra of Transformed Samples

**Figure 5:**
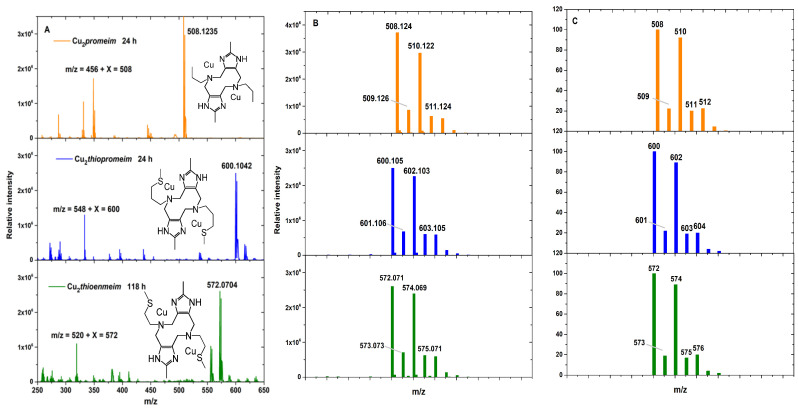
ESI-MS spectra at the end of the transformation: (**A**) full spectra, (**B**) enlargement of the main peak, and (**C**) calculated isotopic distribution. Above, Cu_2_*promeim*; middle, Cu_2_*thiopromeim* and below, Cu_2_*thioenmeim*. Inset structures schematically describe the species with respective masses of 456, 548, and 520 mass units.

Transformation of the three complexes in acetonitrile solutions was also monitored at intervals by mass spectrometry until no further variations were observed: 24 h for Cu_2_*promeim*, 24 h for Cu_2_*thiopromeim*, and 118 h (ca. 5 days) for Cu_2_*thioenmeim*. The final main peaks for the three complexes were respectively 508.13, 600.1, and 572.07 *m*/*z*. All of them correspond to dinuclear species, as shown by the isotopic pattern in the simulated spectra (Figure 5). Remarkably, these masses correspond to an additional 52 u upon each of the formulae [Cu_2_*promeim*], [Cu_2_*thiopromeim*], and [Cu_2_*thioenmeim*], which have calculated masses of 456, 548, and 520 *m*/*z*, respectively. The 52 u could correspond to 2OH + H_2_O (originating from the hydrated Cu salts employed as the starting material). These findings suggest that the three complexes follow the same path, with an analogous final species, responsible for the blue color observed in all cases.

#### 2.2.5. Kinetics of the Transformation of Cu_2_thiopromeim

**Figure 6:**
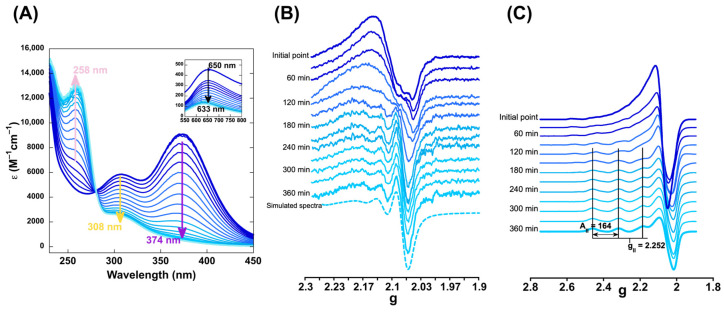
Kinetics of the transformation of [Cu_2_*thiopromeim*](ClO_4_)_4_ in MeCN/EtOH as monitored by UV-vis absorption at room temperature and EPR at room temperature and 150 K. (**A**) Time-dependent UV-vis absorption spectra of the transformation of the [Cu_2_*thiopromeim*] complex in MeCN/EtOH; inset shows the decrease in the ligand field transition. Time-dependent EPR spectra of the transformation of the [Cu_2_*thiopromeim*] complex in MeCN/EtOH at room temperature (**B**) and 150 K (**C**).

Given its intermediate transformation time, Cu_2_*thiopromeim* was chosen to monitor the changes observed over time (in MeCN/EtOH (80%:20%)) in its electronic and EPR spectra (Figure 6).

The ligand field transition at 650 nm, the S → Cu LMCT at 370 nm and the imidazole → Cu LMCT at 308 nm decreased with time (Figure 6A). The intensity changes of these LMCT bands can be analyzed using a simple exponential fit, yielding a rate of decay that is comparable to that of the appearance of the new electronic transition at 258 nm.

The transformation was monitored simultaneously by EPR at room temperature (Figure 6B), showing evident changes in the spectrum over time, likely reflecting changes in Cu(II) coordination. In fact, the decay in EPR intensity at 3132 Gauss can be fit to a rate that is in the same order of magnitude as that of the decay of the LMCT bands observed by absorption (Figure S5), strongly suggesting that the loss of the dinuclear Cu(II) features correlates with the appearance of the 258 nm band and the changes in metal coordination detected by room temperature EPR. At the end of the transformation, the EPR spectrum at room temperature could be simulated (Figure 6B, dotted blue line) by considering only a mononuclear Cu(II) center with limited mobility and a correlation time (t_R_) of 0.18 ns with g_x_ = g_y_ = 2.077, g_z_ = 2.254, A_x_ = A_y_ = 6.79 × 10^−4^ cm^−1^, and A_z_ = 162 × 10^−4^ cm^−1^. In contrast, the time-resolved EPR data at 150 K reveal the loss of the signals associated with the dinuclear Cu_2_*thiopromeim* complex and the appearance of EPR signals (g_||_ = 2.252 and A_||_ = 164 × 10^−4^ cm^−1^) associated with the mononuclear Cu(II) complex (Figure 6). EPR intensity at 2902 Gauss, which had a major contribution from the dinuclear Cu species, also decayed at a rate in the same order of magnitude as the decay rates for the electronic absorption transitions associated with the Cu(II) dinuclear species (see Figure S6). Moreover, after 180 min of reaction, no further spectroscopic changes were observed; the EPR spectrum at 150 K was dominated by a mononuclear species, and spin quantitation confirmed a loss of about half of the EPR intensity (Figure S8). Overall, these data are consistent with the notion that one of the Cu(II) ions in the dinuclear complex undergoes reduction to Cu(I).

#### 2.2.6. Electrochemical Measurements

**Figure 7:**
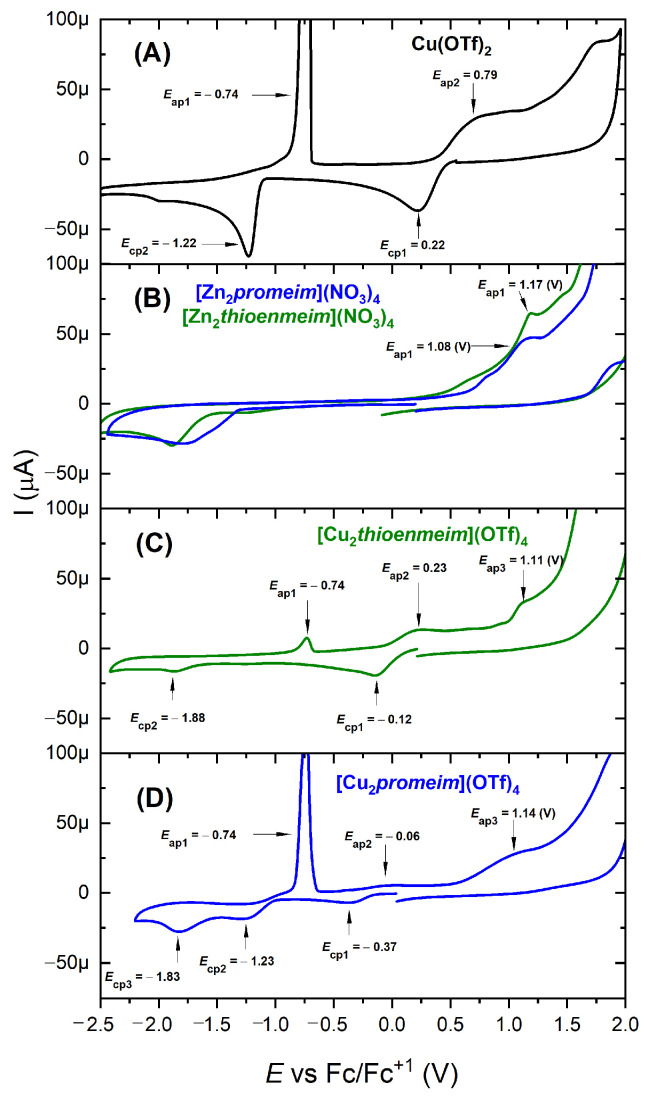
Comparison of cathodic CVs for (**A**) Cu(OTf)_2_; (**B**) [Zn_2_promeim](NO_3_)_4_ and [Zn_2_thioenmeim](NO_3_)_4_; (**C**) freshly prepared [Cu_2_thioenmeim](OTf)_4_ (prior to electron transfer); (**D**) aged [Cu_2_promeim](OTf)_4_ (after electron transfer). All measurements at 0.1 mM in acetonitrile.

Electrochemical studies have provided relevant information in deciphering the electron transfer processes responsible for the spectroscopic changes described above. Given the different times at which each of the three systems undergoes the transformation, electrochemical measurements were used to confirm, firstly, the initial composition of the 2Cu:1 ligand systems, and secondly, their final composition. The electrochemical characterization of the initial state was achieved through CVs of the most inert system, (Cu_2_*thioenmeim*), while for the characterization of the final state, the most labile one, (Cu_2_*promeim*), was examined. All solutions were prepared in MeCN using Cu(OTf)_2_ and the corresponding ligands in a 2Cu:1ligand ratio. Cathodic scan CVs are shown in Figure 7. A MeCN solution of Cu(OTf)_2_ was used as a reference for uncomplexed Cu, while, given the insolubility of the ligands in MeCN, solutions of [Zn_2_*promeim*](NO_3_)_4_ and [Zn_2_*thioenmeim*](NO_3_)_4_ served as references for the ligands unbound to Cu.

In Figure 7A, the reduction wave at 0.22 V corresponds to Cu(II) → Cu(I) and the one at −1.22 V to Cu(I) → Cu°. The corresponding oxidation for Cu° → Cu(I) is observed at −0.74 V and for Cu(I) → Cu(II) at 0.79 V. In this case, the soluble copper species were the simple acetonitrile-solvated copper ions, namely [Cu(MeCN)_4_]^+^ and [Cu(MeCN)_n_]^2+^. In Figure 7B, the CVs of Zn_2_*promeim* and Zn_2_*thioenmeim* show signals ascribable to oxidation of the ligand, at 1.08 and 1.17 V.

The CV of a freshly prepared Cu_2_*thioenmeim* solution (Figure 7C) shows only two reduction waves: one ascribed to Cu(II) → Cu(I) at −0.12 V and that for Cu(I) → Cu° at −1.88 V. The corresponding oxidation waves are Cu° → Cu(I) at −0.74 V and Cu(I) → Cu(II) at −0.23 V. Finally, Figure 7D shows the CV for Cu_2_*promeim* measured approximately one hour after solution preparation, providing enough time for the transformation to reach its final state. In this case, three reduction waves can be observed: the first at −0.37 V, the second at −1.23 V, and the third at −1.83 V (Figure 7D).

Since the Cu(II)/Cu(I) pair is quasi-reversible in all cases, we were able to approximate the E_1/2_ values from the average (E_ap2_ + E_cp1_)/2. These values are: 0.505 V for the solvated Cu ions, 0.055 V for Cu_2_*thioenmeim*, −0.192 for Cu_2_*thiopromeim*, and −0.215 V for the Cu_2_*promeim* copper complexes (Figure S10). The trend in these potentials agrees with expected values for Cu(II)/Cu(I) pairs in the various coordination environments. The largest value, found for the MeCN-solvated ions, is consistent with the great stability known for [Cu(MeCN)_4_]^+^. Concerning the other two copper solutions, it is well documented that copper complexes with sulfur-containing ligands display higher E_1/2_ values than those with exclusively nitrogen-donating ligands [48,49,50,51]. The comparative E_1/2_ values obtained for the studied complexes support the notion that copper ions in the presence of the *thioenmeim* ligand have a N_2_S + _n_MeCN coordination environment, while the coordination environment for copper ions in the presence of the *promeim* ligand is N_2_ + _m_MeCN. The signal observed at the lowest potential values for 2Cu:L systems (E_cp3_ = −1.83 V for L = *promeim* and E_cp2_ = −1.88 V for L = *thioenmeim*) corresponds to the Cu(I) → Cu° reductions. Most revealing is E_cp2_, observed in the 2Cu:*promeim* system at −1.23 V, since it coincides with E_cp2_ for the Cu(OTf)_2_ solution in MeCN, assigned above to [Cu(MeCN)_4_]^+^ → Cu°. This indicates the presence of [Cu(MeCN)_4_]^+^, which is consistent with the isolation of [Cu(MeCN)_4_]OTf crystals from aged similar solutions (Figure S1). It is also of interest to take note of the relative intensities of the first reduction peaks for Cu_2_*promeim* and Cu_2_*thioenmeim*, which were prepared with the same concentrations of ligand and Cu(OTf)_2_ and can be assumed to have similar diffusion coefficients. The marked decrease observed for this signal in Cu_2_*promeim* compared to Cu_2_*thioenmeim* agrees with a marked decrease in the concentration of Cu(II) in the former, which had already been reduced to a large extent at the time of measurement. Finally, in the CV for both Cu complexes, a third oxidation wave can be observed at ca. 1.1 V, attributed to the oxidation of the ligands, as supported by CV measurements performed on the analogous Zn_2_L complexes (Figure 7B).

#### 2.2.7. UV-Vis Spectroelectrochemical Studies on [Zn_2_promeim]^4+^

The last-mentioned oxidation signal was decisive in proposing an electron donor for the reduction of Cu(II); the reasoning is outlined here:

-Concomitantly with the decrease in the LMCT band (Imz ⟶ Cu), we observed a surge in a very intense band at 258 nm. (Figure 3A–C).

-In all three copper complexes, cyclic voltammetry in acetonitrile showed an oxidation signal at ca. 1.2 V, ascribable to an electron loss in the ligand (Figure 7C,D).

-To prove this assignment, we performed cyclic voltammetry on the analogous Zn complex (since the ligand is insoluble in acetonitrile, and Zn(II) is not redox active beyond Zn(II)/Zn°). An oxidation signal was observed at practically the same potential value (Figure 7B).

-We then performed electrochemical oxidation in a UV quartz cell to be able to apply the required potential and simultaneously observe the corresponding spectral changes.

**Figure 8:**
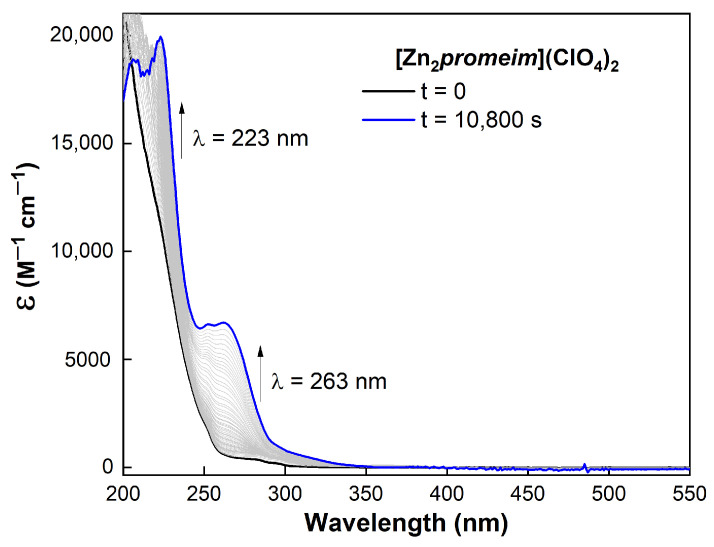
Spectro-electrochemistry of 0.1 mM in situ-generated [Zn_2_*promeim*](ClO_4_)_4_ with 0.1 M NBu_4_PF_6_ as the supporting electrolyte in acetonitrile. The working electrode was Pt mesh, the reference electrode was Ag/AgCl, and a Pt wire served as the counter electrode. Applied potential: 1.1–1.4 V.

-An absorption band at 263 nm surged with the application of the required potential (1.2–1.4 V). This wavelength value is very close to the band observed for the copper complex. (Figure 8).

-This strongly suggests that the oxidation of the ligand caused the new UV absorption.

-Given the chemical structure of the ligand, the HOMO (from which the electron is removed) is expected to have a significant contribution from the nitrogen atoms, particularly those from the heteroaromatic imidazole group. DFT modeling of the initial form of the complex confirms this assumption (*vide infra*).

This assumption is backed up by the previously determined redox potential for the histidine free radical generated by pulse radiolysis, which was found to be 1.17 V [52]. Also supporting this assignment is the corresponding absorption wavelength for the 2-methylimidazole radical, also generated by pulse radiolysis, which was reported at 285 nm [53].

### 2.3. Participation of Dioxygen

#### 2.3.1. Identification of ROS in Cu_2_promeim Solutions

**Figure 9:**
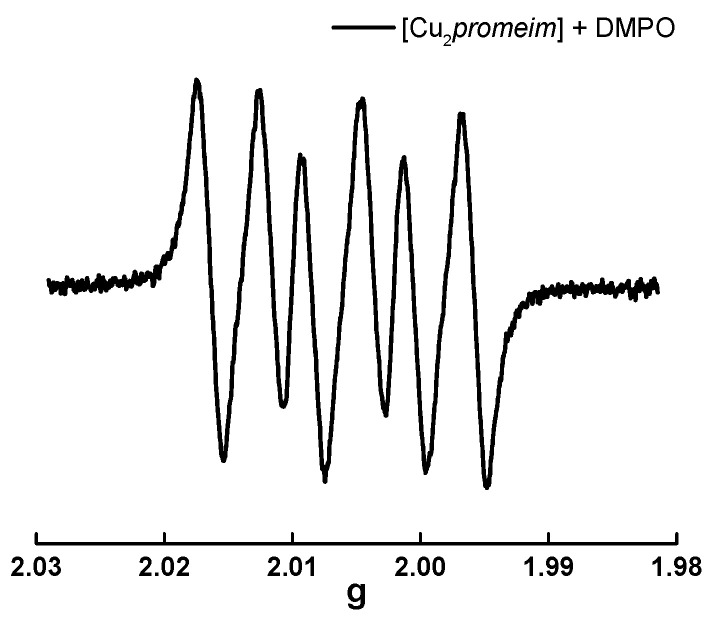
EPR spectrum of the transformation of [Cu_2_*promeim*] in the presence of excess DMPO, in MeCN:EtOH 4:1, 1 mM. The EPR spectrum shows a g = 2.0064 with A_N_ = 1.31 mT, A_Hβ_ = 0.830, A_Hγ_ = was not defined.

Attempting to identify a free radical generated during the reduction of the copper complexes, we recorded the EPR spectrum of Cu_2_*promeim* in MeCN/EtOH in the presence of the spin trap DMPO. The results, shown in Figure 9, agree with the presence of a superoxide radical O_2_^−^ [54,55].

Strikingly similar results were obtained when this measurement was repeated on the same (aerated) solution after different times over several days (Figure S11). In contrast, measuring the spectra of a single sample in a sealed tube showed a marked decrease in the superoxide signal after several hours (Figure S12).

In parallel, the presence of H_2_O_2_ was detected in a MeCN/EtOH solution of Cu_2_*promeim* over a similar period of time, following the method described by Adak et al. [56] (Figure S13). This revealed that, given the availability of dioxygen, the formation of H_2_O_2_ continued for days without signs of coming to an end, providing further evidence of atmospheric oxygen reduction, since the superoxide ion, produced via a one-electron reaction with Cu(I), can subsequently undergo disproportionation to yield peroxide (H_2_O_2_) and molecular oxygen (O_2_), according to the reaction 2 O_2_·^−^ + 2 H^+^ ⟶ H_2_O_2_ + O_2_.

#### 2.3.2. Cu_2_promeim Spontaneous Transformation in the Absence and Presence of Oxygen

**Figure 10:**
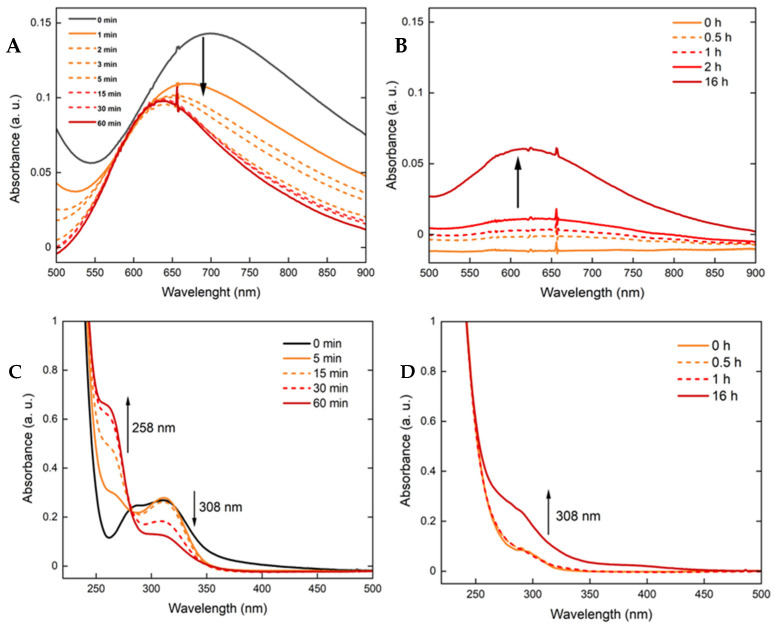
(**A**) Decrease in the d-d absorption for Cu^II^_2_*promeim*. (**B**) Increase in the d-d absorption for Cu^I^_2_*promeim* exposed to O_2_. (**C**) Decrease in the Imz ⟶ Cu LMCT band with simultaneous increase in the new absorption at 258 nm for Cu^II^_2_*promeim*, in the absence of O_2_. (**D**) Increase in the Imz ⟶ Cu LMCT band when Cu^I^_2_*promeim* is exposed to O_2_. Solutions in dry MeCN/EtOH (4:1) were handled under Ar; concentration of Cu_2_*promeim* was 0.2 mM and 1mM for UV and vis regions, respectively.

These results motivated us to compare the transformation of Cu_2_*promeim* in the absence and presence of dioxygen, followed by UV-vis spectroscopy. In Figure 10, we show the results of parallel experiments, in one case (A and C) starting from Cu(II), and in the other (B and D) starting from Cu(I).

When starting from Cu^II^_2_*promeim* in the absence of O_2_, there was a slight blue shift and significant reduction in absorbance, which can be interpreted as a partial reduction of the Cu(II) ions, giving rise to the mixed-valence species Cu^I^Cu^II^*promeim.* In contrast, when the measurement was performed starting from Cu^I^_2_*promeim*, the absorbance of the surging d-d band after exposure to O_2_ hardly reached half of the value of the initial Cu^II^_2_*promeim*, suggesting the permanence of the mixed-valence species Cu^I^Cu^II^*promeim*, in an aerobic medium.

### 2.4. DFT Modeling of the Spontaneously Reduced Form of Cu_2_promeim

Density functional theory (DFT) calculations were carried out to model Cu_2_*promeim*, before and after intramolecular electron transfer. The optimized structure of the starting species comprises the dinuclear complex Cu^II^_2_*promeim* in the usual chair conformation for these systems [35,36,37,38,39,40], in which each Cu(II) is coordinated to a heterocyclic nitrogen from an imidazole group and an aliphatic nitrogen from a tertiary amine. Coordination number five for both copper ions is completed with three acetonitrile molecules, two water molecules, and a hydroxo ion. In the optimized structure for the final state, one of the copper atoms maintained its original coordination environment, but many changes were found for the second site. The oxidation state was 1+ with a coordination number of 3, as determined by XAS. It was bonded to a water molecule, a MeCN molecule and a nitrogen atom from the imidazole ring, which had lost an electron to copper and became a radical, consistent with the spectroelectrochemical study. DFT modeling of this species with a radical on the imidazole ring only succeeded when this group was considered deprotonated.

**Figure 11:**
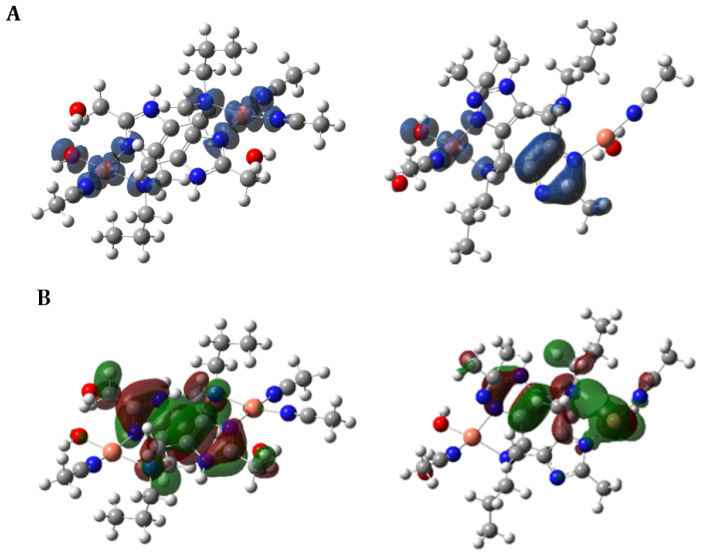
(**A**): Spin density plots for the optimized structures of the initial (**left**) and final species (**right**). (**B**): HOMOs (first doubly occupied molecular orbital) for the complex in its initial (**left**) and final (**right**) state.

Taking all of this into account, the molecular mass for this species agrees with the main signal observed in the MS after the transformation (*vide supra*). Keeping in mind that MeCN molecules escape copper coordination during ESI, this mass corresponds to Cu_2_*promeim* + 52 mass units, which can be accomplished with Cu_2_*promeim*-H^+^ (from the imidazole deprotonation) + 2 H_2_O + OH^−^. Figure 11A displays spin density plots for the optimized structures of the initial and final species, before and after electron transfer. In the former (left), the spin densities are symmetrically distributed among both Cu(II) atoms. In the latter (right), the second unpaired electron is clearly localized above and below the imidazole plane, indicating that an electron transfer occurred from the imidazole ring towards the Cu atom, which underwent reduction, leaving the unpaired electron in the π system of imidazole. The other Cu(II) atom remained unchanged. Figure 11B shows the HOMOs (the first doubly occupied molecular orbital) for the complex in its initial (left) and final (right) state. In the initial complex, the HOMO involved the π system of imidazole moieties. However, after electron transfer, it underwent a dramatic change, involving instead the σ bonding of the Cu(I) center with the imidazole.

During modeling of inner-sphere electron transfer, we consistently found that radical localization on the imidazole moiety was only feasible after deprotonation. Multiple attempts to optimize a structure containing a reduced Cu center and a neutral imidazole radical failed to converge to a stable stationary point; however, optimization became straightforward once deprotonation was introduced. This result indicates that deprotonation is a prerequisite for the subsequent electron transfer step.

These calculations represent the outcome of an extensive exploration, which included the evaluation of several DFT functionals, consideration of alternative ligand configurations, and a search for mechanistic pathways with a physically reasonable free-energy profile. For clarity and balance, we present here only the final results most relevant to the experimental observations since the primary aim of this work was experimental, with theoretical modeling included to support and rationalize the findings.

## 3. Discussion

So far, the spectroelectrochemical experiment backs up the proposal that the UV signal at ca. 260 nm is associated with an oxidation in the ligand. Given the structure of these ligands and considering the nature of the HOMO in Cu_2_*promeim* (Figure 11B), the leaving electron must come from the imidazole moiety, producing a free radical. In the copper complexes, the appearance of this UV signal is concomitant with the reduction of one Cu(II), while in the Zn analog, the signal surges when a potential of ca. 1.2 V is applied. DFT modeling supports an intermediate in which the loss of a proton accompanies the loss of the electron from the imidazole moiety, while the coordinated copper atom is reduced.

In contrast, a surprising observation was made when measuring EPR spectra in the presence of DMPO. Superoxide ion O_2_^·−^ was detected at various times over several days.

Likewise, peroxide was detected chemically with KI during the same period, presumably from the disproportionation of superoxide. Figure S13 shows the formation of H_2_O_2_ (detected as I_3_^−^) over three days in the same fully oxygenated solution. This indicates that, as long as dioxygen is available, H_2_O_2_ will continue to accumulate via partial disproportionation of the superoxide ion.

**Scheme 2:**
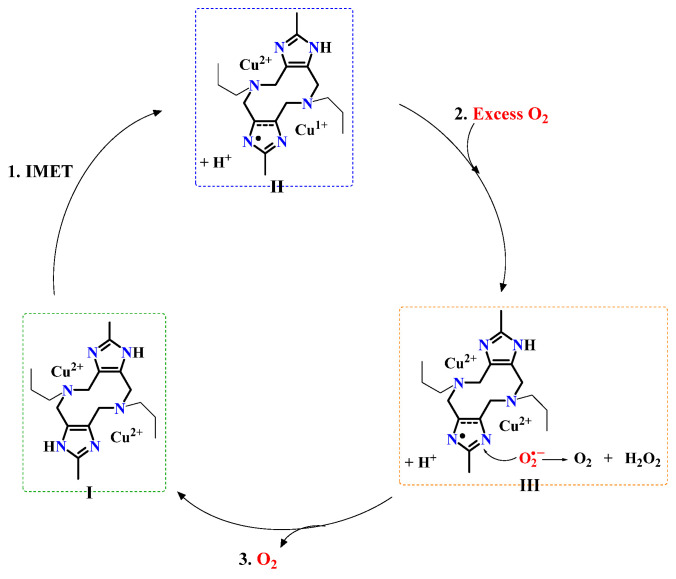
Proposed cycle involving electron transfer between copper, ligand, and dioxygen.

The crucial observation is that, given that O_2_^·−^ is the product of the reaction of Cu(I) with fully available dioxygen, its presence at any given moment can only be explained by assuming that Cu(I) does not completely run out after long periods of time, even when the reaction is considered complete; i.e., it is being replenished. This would mean that as soon as Cu(I) is oxidized by dioxygen, the generated Cu(II) is re-reduced. The only available reducing agent for Cu(II) is the ligand, which was the initial electron donor; however, to perform this task, it would have to be regenerated from its oxidized form, putatively Imz·. The only available reducing agent for Imz· is the superoxide ion O_2_^·−^ [57,58,59,60,61], which is generated at the Cu center. This proposal can be seen more clearly in Scheme 2.

Species I schematically represents the original complex, formed in situ upon mixing the copper salt and the ligand solutions. It rapidly converts to Species II, the nature of which is supported by the following:

-The persistence of the UV absorption at 258 nm, which spectroelectrochemistry assigns to an oxidation of the ligand.

-MS data confirming the dinuclear nature of the complex even after long reaction times.

-XAS measurements detecting the presence of Cu(I) (and Cu(II)) also after long reaction times.

-EPR spectra obtained after the transformation typical of a complex with a single Cu(II).

-Spin quantification after the transformation (Figure S8).

-The persistence of the d-d absorption at the end of the transformation, indicating the presence of Cu(II).

-The coincidence of the final d-d bands observed after Cu^II^_2_*promeim* transforms under Ar and after Cu^I^_2_*promeim* encounters O_2_.

-DFT modeling.

Step 1 in the proposed mechanism consists only of an intramolecular electron transfer, so it must be virtually instantaneous. Step 2, however, involves a bimolecular reaction (between the copper complex and O_2_), which presumably is not as fast as the first step, thus allowing the presence of Species II to be detected. Step 3 also consists of only an intramolecular electron transfer, with O_2_^·−^ considered as part of the coordination center, so it is also expected to be faster than Step 2.

## 4. Materials and Methods

Reagents and solvents used were of reagent quality, purchased from MERCK Sigma Aldrich de México S de RL de CV, Toluca, México *Caution!* Perchlorates are explosive, handle with care.

### 4.1. Synthesis of the Ligands

*Promeim* [62] (2,8-Dimethyl-5,11-dipropyl-1,4,5,6,7,10,11,12 octahydroimidazo [4,5-h]-imidazo [4,5-c][1,6]diazeeine): First, 4.1 g (0.05 mol) of 2-methylimidazole and 2.95 g (0.05 mol) of n-propylamine were added to 20 mL of distilled water. To this mixture, 9.4 mL of a 35% formaldehyde solution (0.12 mol) was added slowly with stirring. The pH was adjusted to 11–12 with concentrated KOH. The mixture was refluxed for 24 h and then stirred for 3 days. The first microcrystalline white precipitate was filtered off and washed with cold water. The remaining solution was allowed to evaporate slowly. After two weeks, a second crop of a crystalline product was isolated, washed with cold water, and dried in air. Total yield: 5.8 g (70%). IR-ATR ν (cm^−1^): 3155, 2963, 2930, 2872, 2249, 1654, 1600, 1531, 1470, 1447, 1412, 1374, 1354, 1344, 1302, 1264, 1218, 1165, 1126, 1059, 1033, 1001, 962, 923, 837, 769, 746, 681, 645, 553, 505, 451, 426, 418. MS-DART (MeOH) *m*/*z*: 331 [C_18_H_30_N_6_] + H^+^. ^1^H-NMR (400 MHz, CD_3_OD): δ = 3.37 (s, 8H), 2.79 (t, *J* = 6.9 Hz, 4H), 2.35 (s, 6H), 1.68 (m, 4H), 1.02 (t, 6H). ^13^C NMR (400 MHz, CD_3_OD): δ 146.31 (NCN imidazole), 59.13 (NCH_2_ propyl), 46.51 (NCH_2_ diacezine), 22.53 (CH_2_ propyl), 13.57 (CH_3_ imidazole), 12.35 (CH_3_ propyl). Elemental Analysis for C_18_H_30_N_6_∙CH_3_OH∙2.5H_2_O Calculated: C, 55.99%; H, 9.65%; N, 20.62%. Found: C, 56.14%; H, 10.34%; N, 23.77. For this ligand, the crystal structure was previously determined.

*Thiopromeim* [63] (2,8-Dimethyl-5,11-bis(3-(methylthio) propyl)-1,4,5,6,7,10,11,12 octahydrodiimidazo [4,5-c:4′,5′-h][1,6]diazecine): In a round-bottom flask, 1.642 g (20 mmol) of 2-methylimidazole was dissolved in 70 mL of H_2_O with constant stirring. To this solution, 2.24 mL (20 mmol) of 3-(methylthio) propylamine was added, followed by the addition of 4.8 mL (60 mmol) of a 12.33 M solution of formaldehyde. To homogenize the reaction mixture, 30 mL of ethanol was added, and the mixture was brought to a basic pH with 2.78 mL (20 mmol) of triethylamine. The reaction mixture was kept under constant stirring at 75 °C in an oil bath for 72 h. After this time had passed, a creamy white precipitate was observed, which was filtered, washed with H_2_O, and left to dry in the oven for 24 h. Yield: 2.342 g (55.5%). IR-ATR ν (cm^−1^): 3030, 2986, 2915, 2817, 2653, 1668, 1596, 1539, 1447, 1409, 1332, 1042, 1005, 918, 773, 654. MS-DART (MeOH) *m*/*z*: 423 [C_20_H_34_N_6_S_2_] + H^+^. ^1^H-NMR (400 MHz, D_2_O): δ = 3.26 (s, 8H), 2.82 (t, *J* = 7.0 Hz, 4H), 2.56 (t, 4H), 2.24 (s, 6H), 2.02 (s, 6H), 1.83 (m, *J* = 7.2 Hz, 4H). Elemental Analysis for C_20_H_34_N_6_S_2_∙CH_3_OH Theoretical: C, 55.47%; H, 8.42%; N, 18.48%; S, 14.10%. Found: C, 55.08%; H, 8.04%; N, 19.77%; S, 13.31%. For this ligand, the crystal structure was previously determined.

*Thioenmeim* (2,8-Dimethyl-5,11-bis(2-(methylthio) ethyl)-1,4,5,6,7,10,11,12 octahydrodiimidazo [4,5-c:4′,5′-h][1,6]diazecine): In a round-bottom flask, 1.6422 g (20 mmol) of 2-methylimidazole was dissolved in 70 mL of H_2_O with constant stirring. To this solution, 1.86 mL (20 mmol) of 2-2-methylthio-ethylamine and 4.86 mL (60 mmol) of a 12.33 M solution of formaldehyde were added. To homogenize the reaction mixture, 10 mL of ethanol was incorporated into the reaction mixture. Finally, 2.8 mmol triethylamine were added to maintain a basic pH. The yellow solution was kept under constant stirring at 75 °C in an oil bath for 72 h. After this time, a white precipitate was observed, which was filtered, washed with H_2_O, and left to dry in the oven for 24 h. Yield: 1.41 g (35%). IR-ATR ν (cm^−1^): 3028, 2968, 2909, 2832, 2661, 1669, 1600, 1538, 1449, 1412, 1223, 1163, 1105, 1046, 965, 918, 849, 836, 787, 754, 688, 652, 630. MS-DART (MeOH) *m*/*z*: 395 [C_18_H_30_N_6_S_2_] + H^+^. ^1^H-NMR (400 MHz, D_2_O): δ = 3.29 (s, 8H), 2.96 (t, *J* = 6.9 Hz, 4H), 2.70 (t, *J* = 6.9 Hz, 4H), 2.25 (s, 6H), 2.07 (s, 6H). ^13^C NMR (400 MHz, CD_3_OD): δ 146.32 (NCN imidazole), 56.70 (NCH_2_ methylene), 46.58 (NCH_2_ diacezine), 33.79 (SCH_2_ methylene), 15.58 (SCH_3_ t), 13.59 (CH_3_ imidazole). Elemental Analysis for C_18_H_30_N_6_S_2_ Calculated: C, 54.79%; H, 7.66%; N, 21.30%; S, 16.25%. Found: C, 54.07%; H, 7.52%; N, 21.92%; S, 15.86%.

### 4.2. Copper Complexes Synthesis and Characterization

[Cu_2_*promeim*(H_2_O)_6_](NO_3_)_4_: In a round-bottom flask, 0.241 g of Cu(NO_3_)_2_·3H_2_O (1.0 mmol) was dissolved in 25 mL of methanol under constant stirring. To this solution, 0.192 g (0.5 mmol) of the *promeim* ligand was added stepwise. At the end, a blue-green solution was obtained, which was rotavaporated to remove the solvent. After complete dryness, the glassy solid was washed with diethyl ether. A blue-green glassy solid was isolated. Yield: 0.386 g (95%). IR-ATR ν (cm^−1^): 3541, 3298, 2979, 2881, 1755, 1714, 1657, 1580,1551, 1493, 1460, 1410, 1276, 1151, 1067, 1039, 1006, 933, 880, 848, 813, 771, 747, 710, 700, 674, 557, 509, 479, 452, 425. MS-ESI (MeOH) *m*/*z*: 454.8 [C_18_H_30_Cu_2_N_6_]-2H^+^. UV-vis MeOH solution 1 mM: λmax, ε (nm, M^−1^ cm^−1^): 274, 2900; 747, 70. Elemental Analysis for C_18_H_42_Cu_2_N_10_O_18_ Calculated: C, 26.57%; H, 5.20%; N, 17.21%. Found: C, 26.62%; H, 4.96%; N, 17.77.

[Cu_2_*thiopromeim*(H_2_O)_2_(NO_3_)_2_](NO_3_)_2_: The synthesis of the complex was performed by dissolving 0.241 g of Cu(NO_3_)_2_·3H_2_O (1.0 mmol) in 25 mL of methanol under constant stirring. First, 0.211 g (0.5 mmol) of *thiopromeim* was added stepwise to the copper salt-containing solution. The mixture turned cloudy, so approximately 5 mL of H_2_O was added to homogenize the solution. Finally, an emerald-green solution was obtained, which was left at room temperature to evaporate the solvent. After a few days, the formation of emerald green crystals was observed. Yield: 0.3875 g (94%). IR-ATR ν (cm^−1^): 3540, 3191, 3128, 3074, 3015, 2973, 2936, 2837, 2819, 2723, 1735, 1636, 1552, 1450, 1435, 1393, 1375, 1348, 1322, 1279, 1174, 1155, 1077, 1071, 1063, 1040, 1023, 1008, 978, 967, 921, 880, 861, 845, 822, 809, 789, 737, 720, 697, 684, 638, 597, 565, 534, 509, 484, 466, 444, 427. MS-ESI (MeOH) *m*/*z*: 546 [C_20_H_34_Cu_2_N_6_S_2_]-2H^+^. UV-vis MeOH solution 1 mM: λmax, ε (nm, M^−1^ cm^−1^): 290, 2900; 345, 4100; 693, 190. Elemental Analysis for C_20_H_38_Cu_2_N_10_O_14_S_2_. Calculated: C, 28.81%; H, 4.59%; N, 16.80%; S, 7.69%. Found: C, 29.04%; H, 4.47%; N, 16.76%; S, 7.50%.

[Cu_2_*thioenmeim*(H_2_O)_2_(NO_3_)_2_](NO_3_)_2_: The synthesis of the cupric complex was performed by dissolving 0.241 g of Cu(NO_3_)_2_·3H_2_O (1.0 mmol) in 25 mL of methanol under constant stirring. To this solution, 0.197 g (0.5 mmol) of the *thioenmeim* ligand was added stepwise. The mixture turned cloudy, so approximately 5 mL of H_2_O was added to homogenize the solution. Finally, an emerald green solution was obtained, which was left at room temperature to evaporate the solvent. After a few days, the formation of emerald green crystals was observed. Yield: 0.362 g (91%). IR-ATR ν (cm^−1^): 3393, 3196, 3120, 3073, 3047, 2973, 2948, 1750, 1638, 1551, 1473, 1445, 1391, 1370, 1350, 1298, 1284, 1256, 1201, 1148, 1087, 1072, 1065, 1036, 1018, 984, 946, 916, 879, 846, 829, 811, 787, 743, 721, 710, 682, 656, 638, 591, 505, 463, 426. MS-ESI (MeOH) *m*/*z*: 491 [C_16_H_26_Cu_2_N_6_S_2_]-2H^+^, the loss of the methyl group from the thioether; 518 [C_18_H_30_Cu_2_N_6_S_2_]-2H^+^. UV-vis MeOH solution 1 mM: λmax, ε (nm, M^−1^ cm^−1^): 290, 2750; 334, 4100; 695, 230. Elemental Analysis for C_18_H_34_Cu_2_N_10_O_14_S_2_ Calculated: C, 26.83%; H, 4.25%; N, 17.38%; S, 7.96%. Found: C, 27.08%; H, 4.33%; N, 17.09%; S, 7.83%.

Sample preparation for kinetic monitoring: Since the ligand was insoluble in acetonitrile, it was dissolved in ethanol, and the copper perchlorate was dissolved in acetonitrile. For the UV measurements, the concentrations in the cuvette were 0.4 mM in Cu and 0.2 mM in L, using either *promeim*, *thiopromeim*, or *thioenmeim*. For visible absorption, EPR and MS, the concentrations were 2.0 mM for Cu and 1.0 mM for the ligand. Details on the preparation of the samples are given in the Supplementary Materials.

## 5. Conclusions

The complete set of results obtained from the various techniques (in acetonitrile or in 4:1 acetonitrile/ethanol solutions) strongly suggests that in these complexes, the reduction of one Cu(II) to Cu(I) is indeed an intramolecular electron transfer reaction where the ligand acts as the electron donor, presumably from the imidazole moiety. A new intense band was observed in the UV spectra of the copper complexes undergoing spontaneous reduction, as well as in that of the analogous zinc complex when it was electrochemically oxidized.

While all three complexes undergo equivalent transformations, the different rates at which they reach the “final” state are associated with the initial loss of the thioether–copper bond in Cu_2_*thiopromeim* and Cu_2_*thioenmein*, which produces, at different times, a coordination environment equal to that of Cu_2_*promeim.* It is from this coordination state that the electron transfer proceeds, giving way to the “final” state. The various spectroscopic measurements performed on solutions of these compounds (UV-vis, EPR, MS, XANES), as well as the electrochemical determinations, are consistent with the DFT modeling of the Cu_2_*promeim* complex, which predicts a mixed-valence Cu^I^Cu^II^L· with a free radical on the imidazole moiety of the ligand.

Both the oxidation potentials obtained here for the ligands and the UV absorption wavelength of the transformed species coincide with previous reports for imidazole-derived radicals [52,53]. We propose that the apparent persistence of this mixed-valence complex with a free radical form of the ligand is maintained because it is not a static structure but part of a cyclic equilibrium of very fast reactions involving widely available dioxygen. Cu(I) is initially generated when Cu(II) accepts an electron from the ligand (which, according to DFT modeling, converts into radical Imz· with the loss of a proton) and is immediately reoxidized to Cu(II) by dioxygen, which then forms the superoxide ion. This superoxide ion can donate an electron to radical Imz·. With the reincorporation of a proton, the imidazole group is regenerated, and a new cycle can begin.

Most copper enzymes consistently feature coordinated imidazole residues and are usually involved in proton-coupled electron transfers with the generation of free radicals [64]. It is also known that Cu(I) interacts with molecular oxygen, generating different types of highly reactive copper–dioxygen adducts, including mononuclear end-on Cu(II)-O_2_^·−^ like the one proposed here [65]. We hope that the findings presented here can be relevant in the understanding of the role of this substructure in biological systems.

## Data Availability

The data presented in this study are available on request from the corresponding author.

## Supplementary Materials

The following supporting information can be downloaded at: https://www.mdpi.com/article/10.3390/molecules31081245/s1. Ligand and compound characterization, X-ray diffraction refining data, and crystal packing, EPR simulation parameters, UV-vis titration of *promeim* and *thioenmeim* with Cu(II), spin quantification, anodic VC, superoxide EPR detection over time, H_2_O_2_ chemical detection, DFT cartesian coordinates and total energy of Cu_2_*promeim* before and after transformation [66,67,68,69,70,71,72,73,74,75,76].

## Abbreviations

The following abbreviations are used in this manuscript:

MeCN	Acetonitrile
cp	Cathodic peak
ap	Anodic peak
