# Supplementary material for: Spontaneous Reduction of Cu(II) Complexes with Imidazole-Derived Ligands in Acetonitrile
Source: Molecules. 2026 Apr 9;31(8):1245. doi: 10.3390/molecules31081245 (PMC13118940; doi:10.3390/molecules31081245)
Supplement: Supplementary file 1 [file molecules-31-01245-s001.zip › molecules-4198510-supplementary.pdf]

Supporting Information

Spontaneous reduction of Cu(II) complexes with imidaz-ole-derived ligands.

Brenda Sánchez-Eguía[1*], Carolina Sánchez-López[2], Marcos Flores-Álamo[1], Nils Schuth[3], Víctor M. Ugalde-Saldívar[1], Virginia Gómez-Vidales[4], Chiara E. Campi[5], J. Raúl Álvarez-Idaboy[1], Liliana Quintanar[2,3], and Laura Gasque[1*].

[1.]Facultad de Química, Universidad Nacional Autónoma de México, CDMX, 04510, México. [2.]Center for Research in Aging, Center for Research and Advanced Studies (Cinvestav), CDMX, 14330, México [3.]Department of Chemistry, Center for Research and Advanced Studies (Cinvestav), CDMX, 07350, México. [4.]Instituto de Química, Universidad Nacional Autónoma de México, Circuito Exterior, CU, 04510, México. [5] Institute of Inorganic and Analytical Chemistry, Justus Liebig University Giessen, 35390, Germany.

Email: gasquel@unam.mx, brenataly@comunidad.unam.mx

1 Materials and methods 2

2 Equipment and tools 2

2.1 X-Ray Structure determination 2

2.2 UV-Vis spectroscopy 2

2.3 Mass spectrometry 2

2.4 Combustion analysis 2

2.5 EPR spectroscopy 2

2.6 Sample preparation for Kinetic monitoring 3

2.7 NMR spectroscopy 3

2.8 IR spectroscopy 3

2.9 Electrochemical studies 3

2.10 Spectroelectrochemistry (SEC) 3

2.11 X-ray absorption spectroscopy (XAS). 3

2.12 Computational Details 4

3 Compound characterization 4

3.1 Single Crystal X-Ray Diffraction 5

3.2 Electron paramagnetic resonance 8

3.3 Mass spectroscopy for ligand transformation 9

3.4 Kinetics trace of absorption spectroscopy 10

3.5 UV-Vis titration 10

3.6 Spin quantification 11

3.7 Electrochemistry 11

3.8 Superoxide detection by DMPO over time 13

3.9 UV-Vis indirect detection of H_2O_2 over time 14

3.10 Infrared ATR 15

3.11 Mass spectrometry 17

3.12 ^1H and ^{13}C Nuclear magnetic resonance 20

3.13 DFT calculations 22

4 References 27

1 Materials and methods

Reagents and solvents used were of commercially available reagent quality unless otherwise stated. *Caution!* Perchlorates are explosive, handle with care.

2 Equipment and tools

2.1 X-Ray Structure determination

Suitable single crystals of compounds Cu_2*thiopromeim* and Cu_2*tioenmemim* were mounted on mounted on a glass fiber under cryogenic system and crystallographic data were collected with an Oxford Diffraction Gemini "A" diffractometer with a CCD area detector with $\lambda MoK\alpha$ = 0.71073 Å and monochromator of graphite at 130 K, CrysAlisPro and CrysAlis RED software packages[1] were used for data collection and integration. Analysis of the integrated data did not reveal any decay. Collected data were corrected for absorption effects by an analytical numeric absorption correction[2] using a multifaceted crystal model based on expressions upon the Laue symmetry with equivalent reflections. Structure solution and refinement were carried out with the programs SHELXT[3] and SHELXL[4] respectively; WinGX2021[5] and Mercury software[6] were used to prepare material for publication. Full-matrix least-squares refinement was carried out by minimizing $(Fo^2 - Fc^2)^2$. All nonhydrogen atoms were refined anisotropically. H atoms of the water (O-H) and amine (N-H) groups were located in a difference map and refined isotropically with Uiso(H) of 1.5 Ueq for H-O and 1.2 Ueq for N-H. Hydrogen atoms attached to carbon atoms were placed in geometrically idealized positions and refined as riding on their parent atoms, with C—H = 0.96 – 0.99 Å with Uiso (H) = 1.2Ueq(C) for methylene groups, and Uiso (H) = 1.5 Ueq(C) for methyl group. Crystal data and experimental details of the structure determination are listed in Table S3. Crystallographic data have been deposited at the Cambridge Crystallographic Data Center as supplementary material CCDC: 2442956-2442957. Copies of the data can be obtained free of charge on application to CCDC, 12 Union Road, Cambridge CB2 1EZ, UK. e-mail: deposit@ccdc.cam.ac.uk.

2.2 UV-Vis spectroscopy

The solution behaviour of the cupric complexes was analysed by means of UV-Vis spectroscopy, known concentration solution were prepared in different solvents, first the blank spectra was measured followed by the sample, the measurements were performed either in standard or kinetic mode. UV–visible experiments were carried out with an Agilent spectrophotometer model 8453 equipped with a ChemStation software, using a 1 cm cuvette cell.

2.3 Mass spectrometry

Mass spectra were obtained on a JEOL AccuTOF JMS-T100LC (DART). Electrospray Ionization (ESI) MS were obtained with a Bruker Esquire 6000 spectrometer equipped with ion trap and for kinetics Agilent 6530 ESI-QTOF.

2.4 Combustion analysis

Elemental analyses were obtained with a Thermo Scientific “PerkinElmer” Model 2400 Series II CHNS/O, at a temperature of 950°C using a “Sartorious” model M2P microbalance; cystine, acetanilide and sulfamic acid Thermo Scientific with certification were used as standard.

2.5 EPR spectroscopy

Electron Paramagnetic Resonance (EPR) data were obtained as frozen solutions in quartz tubes at 77 K with a JEOL JES-TE300 equipment at X-band frequency (9.4 GHz), 100 KHz modulation. External magnetic field was measured with a JEOL ESFC5 precision gaussmeter. An ITC controller was employed for VT measurements, data were acquired and manipulated with the ESPRIT-328, v.1-916 software. X-band EPR spectra were collected using either an EMX Plus Bruker System, with an ER 041 XG microwave bridge and an ER 4102ST cavity; or a JEOL JES-TE300 equipment with a JEOL ESFC5 precision gaussmeter. Room temperature EPR spectra were collected using the following parameters: microwave frequency, 9.4 GHz; microwave power, 5.02 mW; modulation amplitude, 2.00 G; modulation frequency, 100.00 kHz; time constant, 327.68 msec; conversion time, 20.00 msec; sweep time 81.84 sec and averaging over 3 scans. At 150 K, spectra were collected using an ER4131VT variable temperature nitrogen system and the following conditions were used: microwave frequency, 9.4 GHz; microwave power, 10.02 mW; modulation amplitude, 5.00 G; modulation frequency, 100.00 kHz; time constant, 327.68 msec; conversion time, 82.00 msec; sweep time 83.97 sec and averaging over 3 scans. EPR spectra were simulated using the program Easyspin 5.2.28 in MathLab.

2.6 *Sample preparation for Kinetic monitoring*

The Cu:L ratio is 2:1; since the ligand is insoluble in acetonitrile, it is dissolved in ethanol, and the copper perchlorate in acetonitrile. For the UV measurements, the concentrations in the cuvette are 0.4 mM in Cu and 0.2 mM in L – *promeim, thiopromeim, thioenmeim*. To achieve these concentrations in the cuvette, the stock Cu solution in acetonitrile is 2 mM, and the stock ligand solution in ethanol is 1 mM. From these stock solutions, 0.5 mL of each is taken and placed in the cuvette containing 1.5 mL of acetonitrile. In this way, the acetonitrile-to-ethanol ratio in the cuvette is 4:1. For the experiment in the presence of base, adequate volume from a 1 M NaOH solution is added to the stock ethanolic solution of the ligand. Since the reaction occurs immediately, the preparation is as follows: 1.5 mL of acetonitrile is first placed in the cuvette with a stirrer bar, followed by 0.5 mL of the Cu solution (also in acetonitrile). Just before starting the measurement, 0.5 mL of the ligand ethanolic solution is added. The measurement is then performed in kinetics mode, monitoring the evolution of the bands (LMCT from imidazole, thioether to Cu) and 258 nm (radical formation). For the visible region (to follow the d-d transition band), EPR and mass spectrometry the concentration is 5 times higher; that is, Cu at 2 mM in acetonitrile and the ligand at 1 mM in ethanol. To achieve these concentrations, it is advisable to prepare stock solutions at 5 mM and perform the necessary calculations, ensuring that the solvent ratio is always maintained.

2.7 *NMR spectroscopy*

NMR spectra were obtained on a 400 MHz Bruker Avance spectrometer, referenced to tetramethylsilane as internal standard, or the residual protonated solvent peak.

2.8 *IR spectroscopy*

IR spectra were acquired on a FTIR / FIR Spectrum 400 Perkin-Elmer spectrometer in the 4000-400 cm^{-1} range.

2.9 *Electrochemical studies*

The experiments were performed in a potenciostat-galvanostat model CHI1760E of CH Instruments with a conventional three-electrode cell composed by a reference electrode Ag/AgCl/0.1 M NH_4Cl in acetonitrile, a platinum wire as auxiliary electrode, and a glassy carbon disk (surface area 7.1 mm^2) as the working electrode. Cyclic voltammetry was performed in the potential range of –2 to 2.8 V at a sweep rate of 200 mV s^{-1} and was initiated from open-circuit potential ($E_i = 0$). Voltammograms were taken for 1 mM solutions of copper complexes in acetonitrile with 0.1 M $[n\text{-}Bu_4N]PF_6$ which played the role of supporting electrolyte. The solutions were purged in the cell with a purified nitrogen stream for 10 min prior to the studies. Potentials were recorded versus a pseudoreference electrode of AgCl(s)/Ag(wire) immersed in 0.1 M $N[n\text{-}Bu_4N]Cl$ acetonitrile. In agreement with IUPAC convention, the voltammogram of the ferrocenium/ferrocene (Fc^+/Fc) system was obtained to establish the values of half wave potentials ($E_{1/2}$) from the expression $E_{1/2} = (E_{pa} + E_{pc})/2$.

2.10 *Spectroelectrochemistry (SEC)*

In a potenciostat-galvanostat model CHI1760E of CH Instruments together with an Agilent spectrophotometer model 8453 equipped with a ChemStation software, a quartz cell cuvette with a stirrer bar was adapted with three electrode arrangement, in order to perform cyclic voltammetry coupled with UV-Vis spectroscopy. NBu_4PF_6 0.1 M was used as supporting electrolyte in acetonitrile, working electrode was Pt mesh, Ag/AgCl reference electrode and counter electrode a Pt wire, the generated in-situ complex, $[Zn_2promeim](ClO_4)_4$ 0.1 mM. The spectroscopic measurements were performed in kinetic mode for 3 h, at the same time the solution was electrolyzed by cyclic sweeps going from a potential range of 1.2 – 1.4 V (values previously established by individual cyclic voltammetry studies) with a slow sweep rate i.a. 0.001 V. The experiment was performed three times to observed reproducibility.

2.11 *X-ray absorption spectroscopy (XAS).*

$Cu(ClO_4)_2$ was dissolved in acetonitrile to yield a 5 mM concentration and mixed with the respective ligands to result in a 2:1 copper:complex stoichiometry and left on the bench for three days. The solutions were injected into a Kapton-sealed acrylic ~100 µl sample holder of our own design, frozen in liquid nitrogen and stored until the analysis. All data were collected at SSRL beamline 9-3, which is equipped with a liquid nitrogen cooled double-crystal monochromator (Si(2,2,0)). The Cu K-alpha emission signal was detected using a Lytle detector shielded from unwanted photons by Soller slits and a Ni foil. The beam spot size on the sample was cut with slits to 0.7 mm vertical and 0.3 mm horizontal. To reduce photo-damage, a liquid He cooled cryostat for measurements at 10 K was used, the beam was attenuated with 100µm thick Al foil, and the sample position was changed after each spectrum. Incoming energies were calibrated against the first inflection point of an internal Cu foil standard at 8980.3 eV.

2.12 Computational Details

The computations were executed utilizing the Gaussian 09 suite of software programs.[7] Geometry optimizations and frequency calculations were conducted employing the 6-31+G(d,p) basis set and the M06 functional[8] with an ultrafine grid coupled with the SMD continuum model.[9] The selection of the M06 functional was founded on its parametrization, which encompasses transition metals and nonmetals, rendering it particularly suitable for organometallic systems.[8] The choice of SMD was predicated on its ability to faithfully replicate solvent effects, with the capability to estimate solvation free energies for both charged and uncharged solutes, featuring relatively minimal inaccuracies. It has been empirically validated as appropriate for mixed models incorporating explicit solvent molecules. Contrary to other continuum models, it has been proven accurate for vibrational calculations in solution.[10] All calculations were executed employing acetonitrile as the solvent. To satisfy all free valences in the complexes containing Copper, coordination sphere around each Cu (II) center, were fulfil in agreement to mass experiments. The remaining solvent was represented through a continuum model.

3 Compound characterization

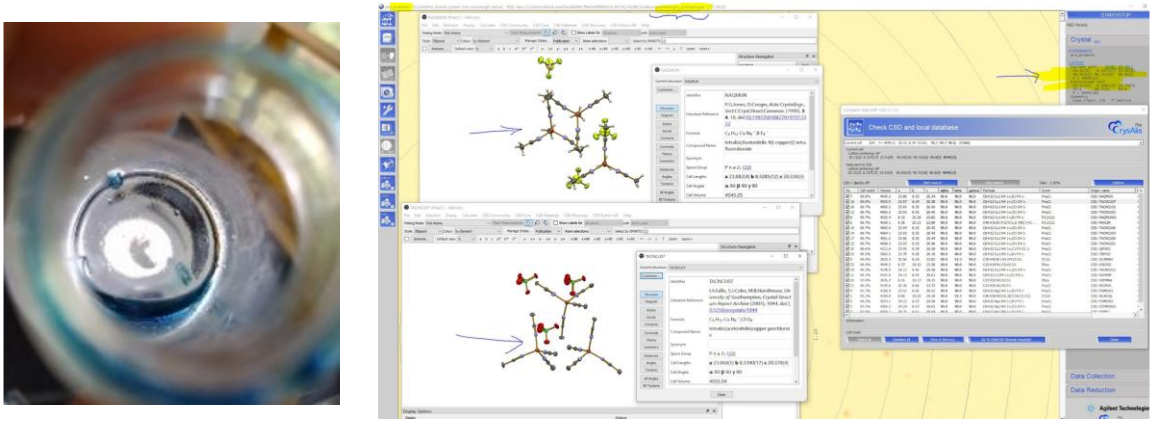

Figure S1. Left. Colourless crystals surrounded by blue solution obtained from concentrated acetonitrile solutions with both *promeim* and *thiopromeim* containing cupric complexes. Right. Crystallographic data collected from those crystals evidenced tetrakis*(*acetonitrile)Cu(I) formation.

3.1 Single Crystal X-Ray Diffraction

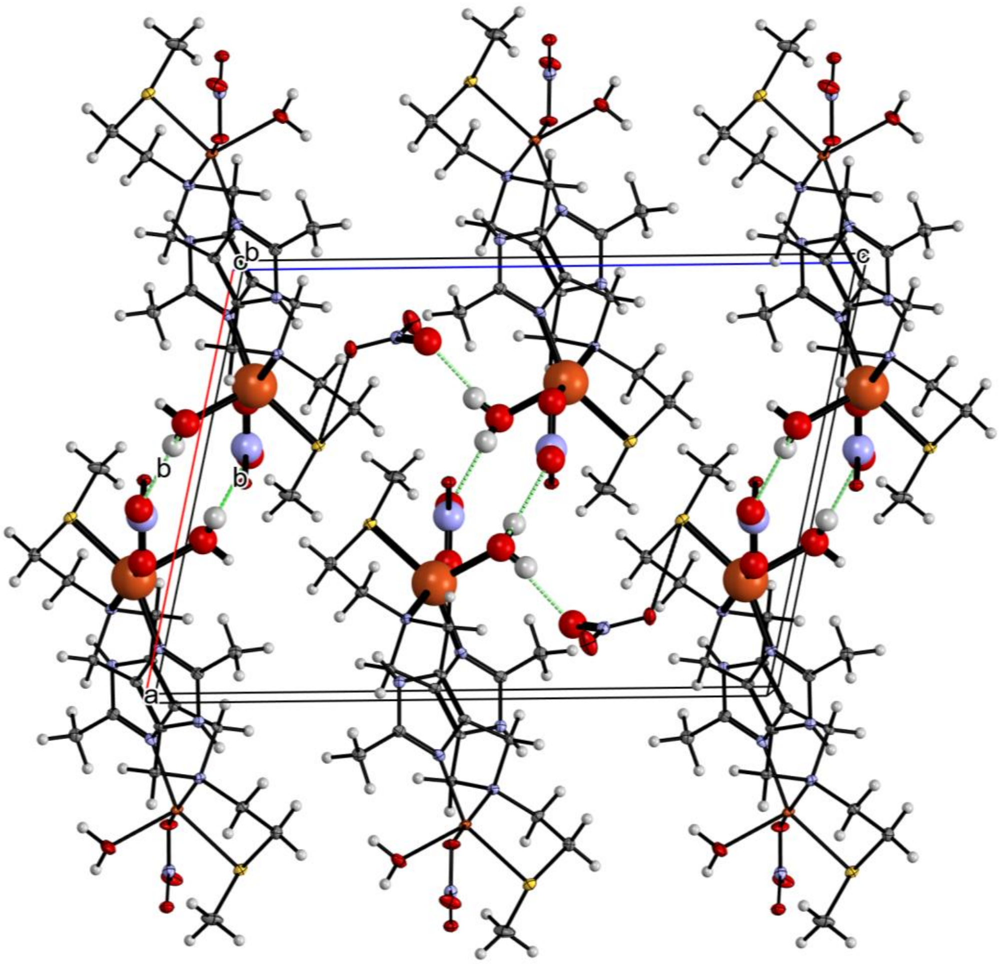

Figure S2-A. View of the intermolecular interactions along the *b* axis of [Cu_2*thioenmeim*(H_2O)$_2$(NO_3)$_2$](NO_3)$_2$, emphasizing the *$R_2^2(12)$, $D_1^1(2)$ motif's*.

In the crystal array of **[Cu_2*thioenmeim*(H_2O)$_2$(NO_3)$_2$](NO_3)$_2$**, intermolecular O—H...O and N—H...O interactions mainly of classical hydrogen bonding are observed. The hydrogen bond O(1W)—H(1D)...O(2) (2.01(4) Å), involving neighboring molecules related by the symmetry operation 1−x, 1−y, −z, generates an *$R_2^2(12)$ motif*. Meanwhile, the interaction O(1W)—H(1E)...O(4) (1.95(3) Å), with symmetry operation x, 1/2−y, −1/2+z, forms a *$D_1^1(2)$ motif* extending along the *a–c* plane. Altogether, these intermolecular interactions give rise to an infinite three-dimensional framework defined by the base vectors [1 0 0], [0 1 0], and [0 0 1].

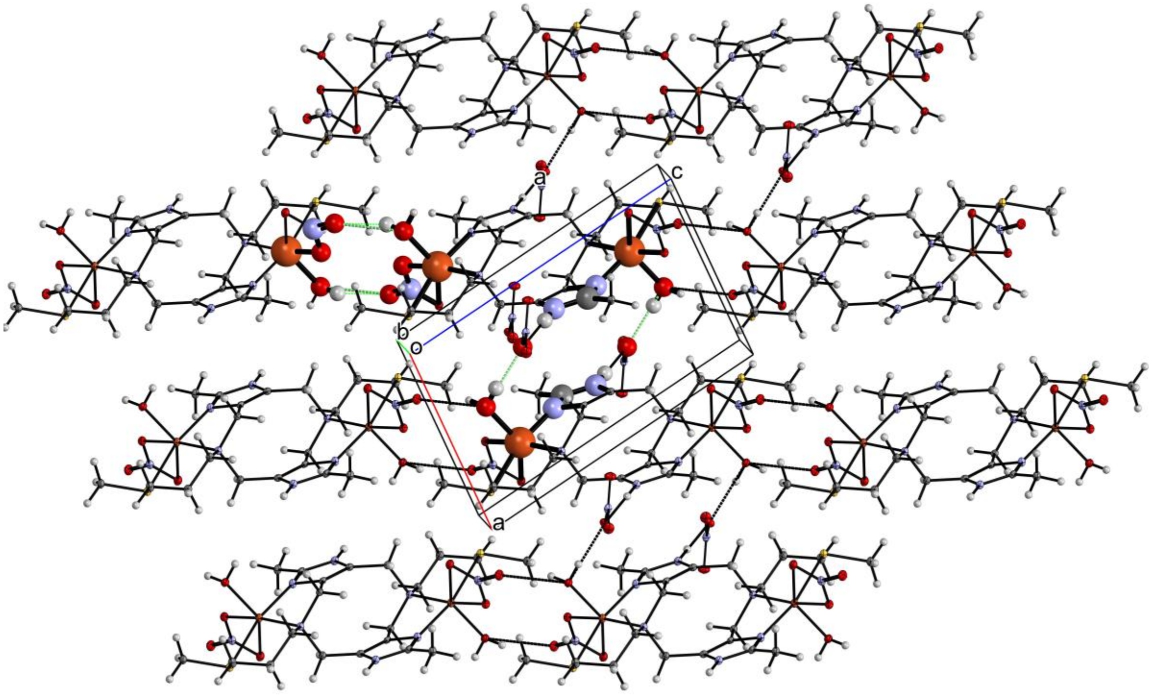

Figure S2-B. View of the intermolecular interactions along the *b* axis of **[*Cu₂thiopromeim*(H₂O)₂(NO₃)₂](NO₃)₂**, emphasizing the *R_4^2(16)* and *R_2^2(12) motif's*.

In the crystal structure of **[Cu_2*thiopromeim*$(H_2O)_2(NO_3)_2](NO_3)_2$**, intermolecular O—H...O and N—H...O interactions of classical hydrogen bonding are observed. The hydrogen bonds O(1W)—H(1E) ...O(5) (1.87(4) Å) and N(3)—H(1F)...O(5) (1.88(4) Å), with symmetry operations x, −1 + y, z and 1 − x, 2 − y, 1 − z, respectively, form a *R_4^2(16) motif*. On the other hand, the interaction weak O(1W)—H(1D)...O(5) (2.12(5) Å), with symmetry code 1 − x, 1 − y, −z, generates an *R_2^2(12) motif.* In summary, these intermolecular interactions show an infinite two-dimensional network defined by the base vectors. [0 0 1] and [1 0 0].

Table S1. Crystal data and structure refinement for compounds [Cu_2*thioenmeim*$(NO_3)_2(H_2O)_2$]NO_3 and [Cu_2*thiopromeim*$(NO_3)_2(H_2O)_2$]NO_3

Identification code	[Cu_2*thioenmeim*$(NO_3)_2(H_2O)_2$]NO_3	[Cu_2*thiopromeim*$(NO_3)_2(H_2O)_2$]NO_3
Empirical formula	$C_{18}H_{34}Cu_2N_{10}O_{14}S_2$	$C_{20}H_{38}Cu_2N_{10}O_{14}S_2$
Formula weight	805.75	833.80
Temperature	130(2) K	130(2) K
Wavelength	0.71073 Å	0.71073 Å
Crystal system	Monoclinic	Triclinic
Space group	P 21/c	P -1
Unit cell dimensions	a = 11.5759(8) Å	a = 7.8707(19) Å
	b = 7.9707(5) Å	b = 9.2355(15) Å
	c = 16.3107(12) Å	c = 11.961(2) Å
	α = 90°.	α = 94.725(13)°.
	β = 102.373(7)°.	β = 96.772(16)°.
	γ = 90°.	γ = 115.16(2)°.
Volume	1470.00(18) $Å^3$	772.9(3) $Å^3$
Z	2	1
Density (calculated)	1.820 Mg/m^3	1.791 Mg/m^3
Absorption coefficient	1.674 mm^{-1}	1.595 mm^{-1}
F(000)	828	430
Crystal size	0.400 x 0.160 x 0.110 mm^3	0.550 x 0.340 x 0.270 mm^3
Theta range for data collection	3.430 to 29.433°.	3.415 to 29.062°.
Index ranges	-15<=h<=15, -10<=k<=7, -22<=l<=21	-10<=h<=9, -11<=k<=12, -15<=l<=15
Reflections collected	8474	8397
Independent reflections	3516 [R(int) = 0.0315]	3617 [R(int) = 0.0653]
Completeness to theta	99.8 %	99.7 %
Refinement method	Full-matrix least-squares on F^2	Full-matrix least-squares on F^2
Data / restraints / parameters	3516 / 3 / 219	3617 / 3 / 228
Goodness-of-fit on F2	1.087	1.039
Final R indices [I>2sigma(I)]	R1 = 0.0399, wR2 = 0.1425	R1 = 0.0610, wR2 = 0.1562
R indices (all data)	R1 = 0.0514, wR2 = 0.1524	R1 = 0.0763, wR2 = 0.1757
Largest diff. peak and hole	0.666 and -0.637 e.$Å^{-3}$	1.250 and -1.131 e.$Å^{-3}$

Table S2. Selected bond lengths [Å] and angles [°] for compounds [Cu_2*thioenmeim*$(NO_3)_2(H_2O)_2$]NO_3 and [Cu_2*thiopromeim*$(NO_3)_2(H_2O)_2$]NO_3

[Cu_2*thioenmeim*$(NO_3)_2(H_2O)_2$]NO_3		[Cu_2*thiopromeim*$(NO_3)_2(H_2O)_2$]NO_3	
Bond	(Å)	Bond	(Å)
C(1)-C(2)	1.520(4)	C(1)-C(2)	1.509(6)
C(1)-S(1)	1.809(3)	C(1)-S(1)	1.814(4)
C(2)-N(1)	1.497(4)	C(3)-N(1)	1.498(5)
C(4)-N(1)	1.494(4)	C(4)-N(1)	1.493(5)
C(5)-C(6)	1.360(4)	C(5)-C(6)	1.361(5)
C(5)-N(2)	1.383(4)	C(5)-N(2)	1.390(5)
Cu(1)-N(2)	1.984(3)	Cu(1)-O(1)	2.008(3)
Cu(1)-O(1)	2.008(2)	Cu(1)-N(2)	2.016(3)
Cu(1)-N(1)	2.127(2)	Cu(1)-N(1)	2.096(3)
Cu(1)-O(1W)	2.161(2)	Cu(1)-O(1W)	2.201(3)
Cu(1)-S(1)	2.3020(9)	Cu(1)-S(1)	2.3403(12)
N(4)-O(3)	1.236(4)	N(4)-O(3)	1.235(5)
N(5)-O(5)	1.238(4)	N(5)-O(7)	1.228(5)
N(5)-O(4)	1.253(4)	C(2)-C(3)	1.507(6)
Angle	(°)	Angle	(°)

N(2)-Cu(1)-O(1)	89.78(10)	O(1)-Cu(1)-N(2)	94.49(13)
N(2)-Cu(1)-N(1)	82.39(10)	O(1)-Cu(1)-N(1)	168.69(12)
O(1)-Cu(1)-N(1)	164.09(10)	N(2)-Cu(1)-N(1)	84.19(13)
N(2)-Cu(1)-O(1W)	100.85(10)	O(1)-Cu(1)-O(1W)	90.11(12)
O(1)-Cu(1)-O(1W)	97.54(10)	N(2)-Cu(1)-O(1W)	101.73(13)
N(1)-Cu(1)-O(1W)	97.53(10)	N(1)-Cu(1)-O(1W)	101.16(12)
N(2)-Cu(1)-S(1)	153.45(8)	O(1)-Cu(1)-S(1)	88.75(9)
O(1)-Cu(1)-S(1)	93.89(7)	N(2)-Cu(1)-S(1)	166.81(11)
N(1)-Cu(1)-S(1)	87.21(7)	N(1)-Cu(1)-S(1)	90.13(9)
O(1W)-Cu(1)-S(1)	104.70(7)	O(1W)-Cu(1)-S(1)	91.03(9)

3.2 Electron paramagnetic resonance

Table S3. EPR parameters from simulation of the [Cu_2*thioenmeim*](ClO_4)$_4$ complex. All hyperfine coupling values are given in MHz.

**Parameter**	**Value**	**Parameter**	**Value**
**g_x (Sys1)**	2.037	**g_x (Sys2)**	2.037
**g_y (Sys1)**	2.096	**g_y (Sys2)**	2.096
**g_z (Sys1)**	2.347	**g_z (Sys2)**	2.347
**A_x (Sys1)**	35	**A_x (Sys2)**	35
**A_y (Sys1)**	30	**A_y (Sys2)**	30
**A_z (Sys1)**	250	**A_z (Sys2)**	250
**g strain$_x$ (Sys1)**	0.030	**g strain$_x$ (Sys2)**	0.030
**g strain$_y$ (Sys1)**	0.027	**g strain$_y$ (Sys2)**	0.027
**g strain$_z$ (Sys1)**	0.032	**g strain$_z$ (Sys2)**	0.032

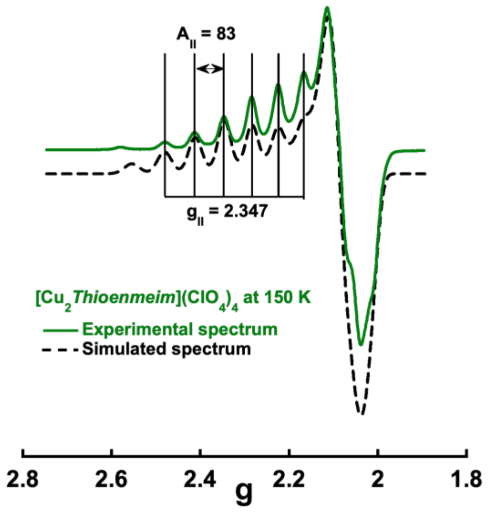

Figure S3. Experimental and simulated EPR of [Cu_2*thioenmeim*](ClO_4)$_4$ complex. EPR simulation (black dashed line) was performed using EasySpin® software (version 6.0.6), using the parameters from table S3.

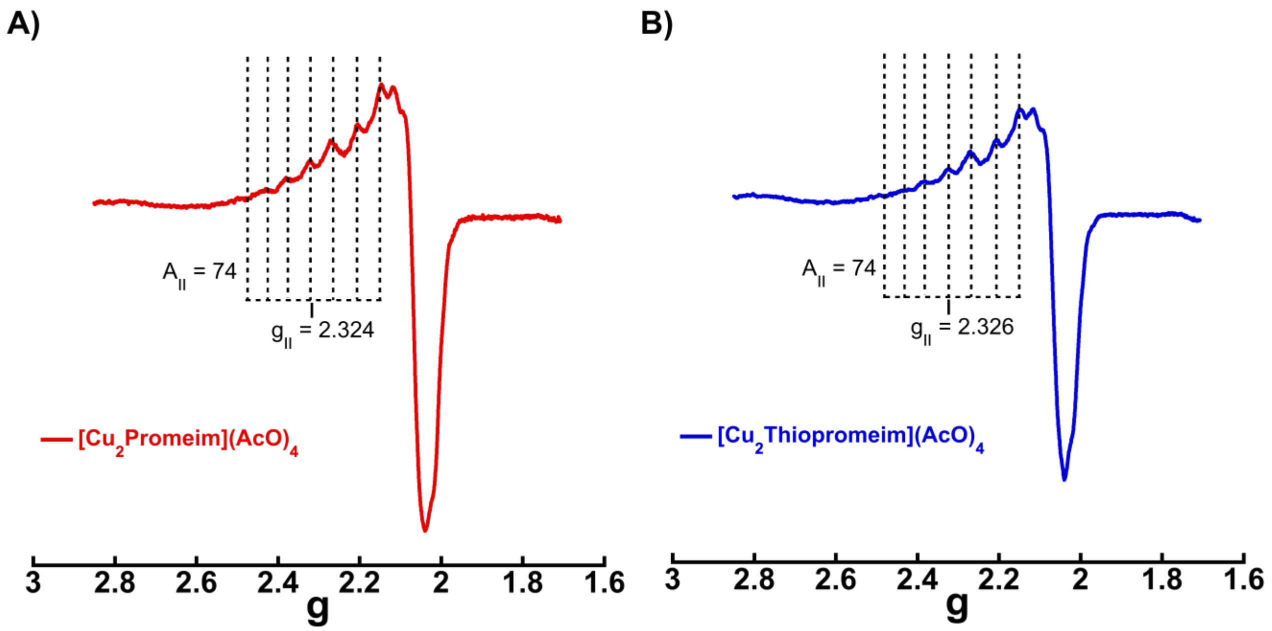

Figure S4. Unpublished preliminary EPR spectra of [Cu_2(*promeim*)]$(AcO)_4$ (A) and [Cu_2(*thiopromeim*)]$(AcO)_4$ (B).

3.3 Mass spectroscopy for ligand transformation

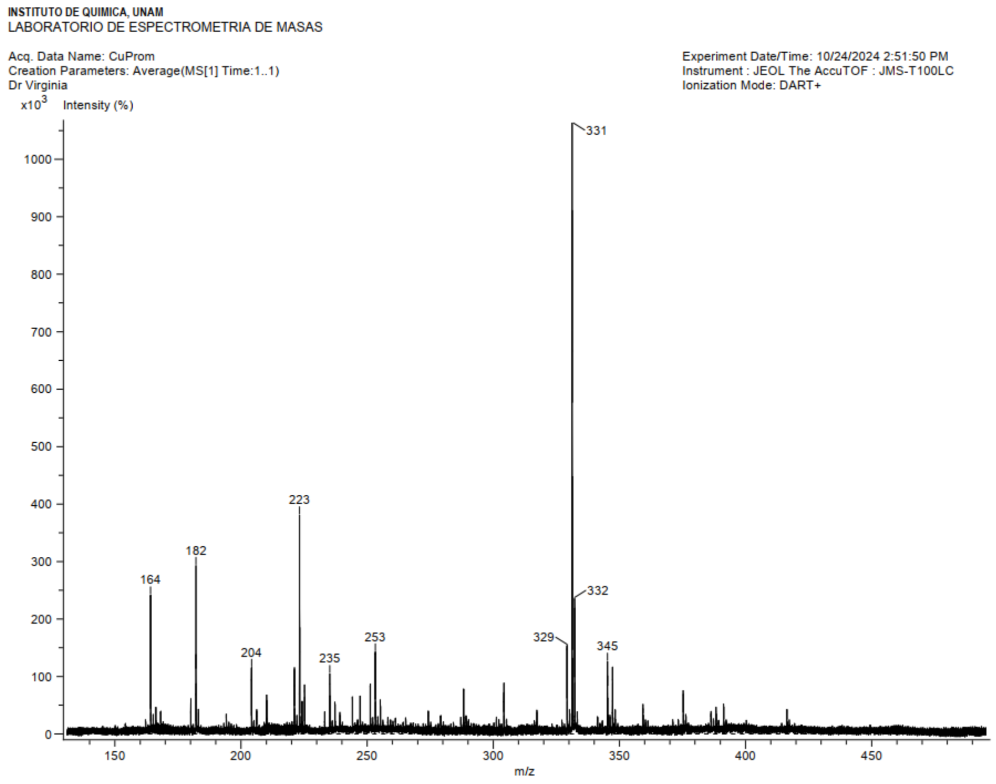

Figure S5. MS of *promeim* ligand after the transformation of the copper complex.

3.4 Kinetics trace of absorption spectroscopy

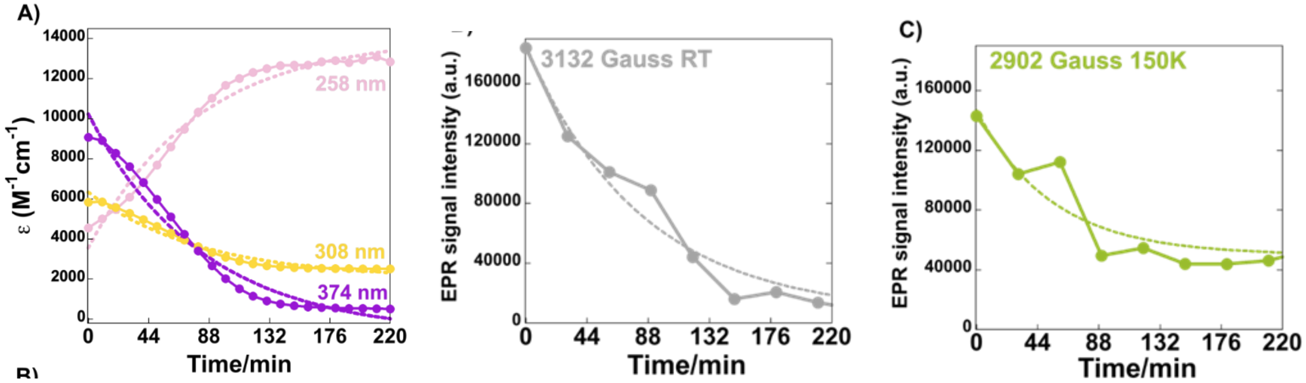

Figure S6. (A) Absorption changes of [Cu_2*thiopromeim*] complex 1 mM as a function of time for the LMCT bands at 308 nm (yellow) and 374 nm (purple) and the electronic transition appearing at 258 nm (pink). LMCT bands seem to follow an exponential decay with a rate of ~0.012 min^{-1}; namely, 0.0122 min^{-1} and 0.01120 min^{-1} for the 308 and 374 nm bands, respectively (yellow and purple traces in (A); quite similar to the rate of appearance of the new electronic transition at 258 nm, i.e. 0.0124 min^{-1} (pink trace in A). EPR spectral changes associated to the transition from a dinuclear to a mononuclear Cu(II) complex: EPR intensity at 3132 Gauss (B at room temperature) decays with a rate of 0.0122 min^{-1}. EPR intensity at 2902 Gauss (C at 150 K), decays with a rate of 0.0184 min^{-1}, which is in the same order of magnitude as the decay rates for the electronic absorption transitions associated to the Cu(II) dinuclear species.

3.5 UV-Vis titration

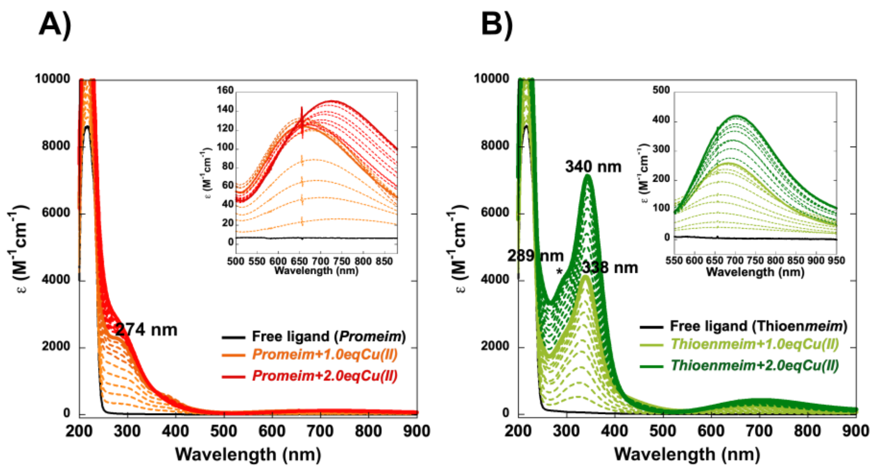

Figure S7. Titration of *promeim* (A) and *thioenmeim* (B) with Cu(II) as followed by UV-Vis electronic absorption. Spectra recorded after addition of 1.0 and 2.0 equiv of Cu(II) are shown in continuous line. The intermediate points 0.1-0.9 and 1.1-1.9 equiv are shown in dashed lines.

3.6 Spin quantification

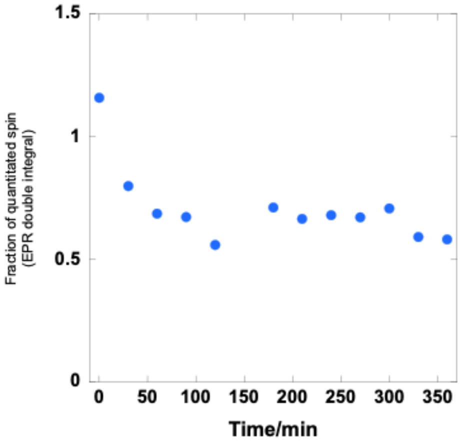

Figure S8. Fraction of quantified spin via EPR double integral as a function of the reaction time of the [Cu_2*thiopromeim*] complex. The calculated fraction is with respect to the initial point of kinetics.

3.7 Electrochemistry

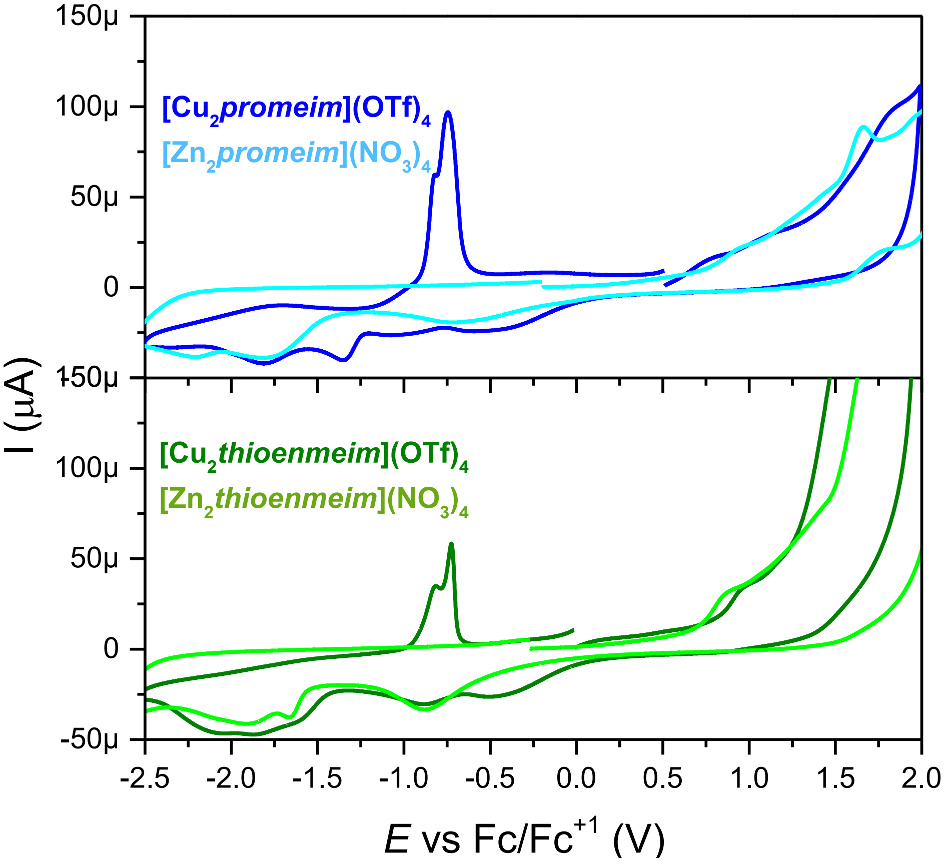

Figure S9. Anodic comparison voltamperograms of Zn and Cu coordination complexes, 1 mM in CH_3CN at 25°C.

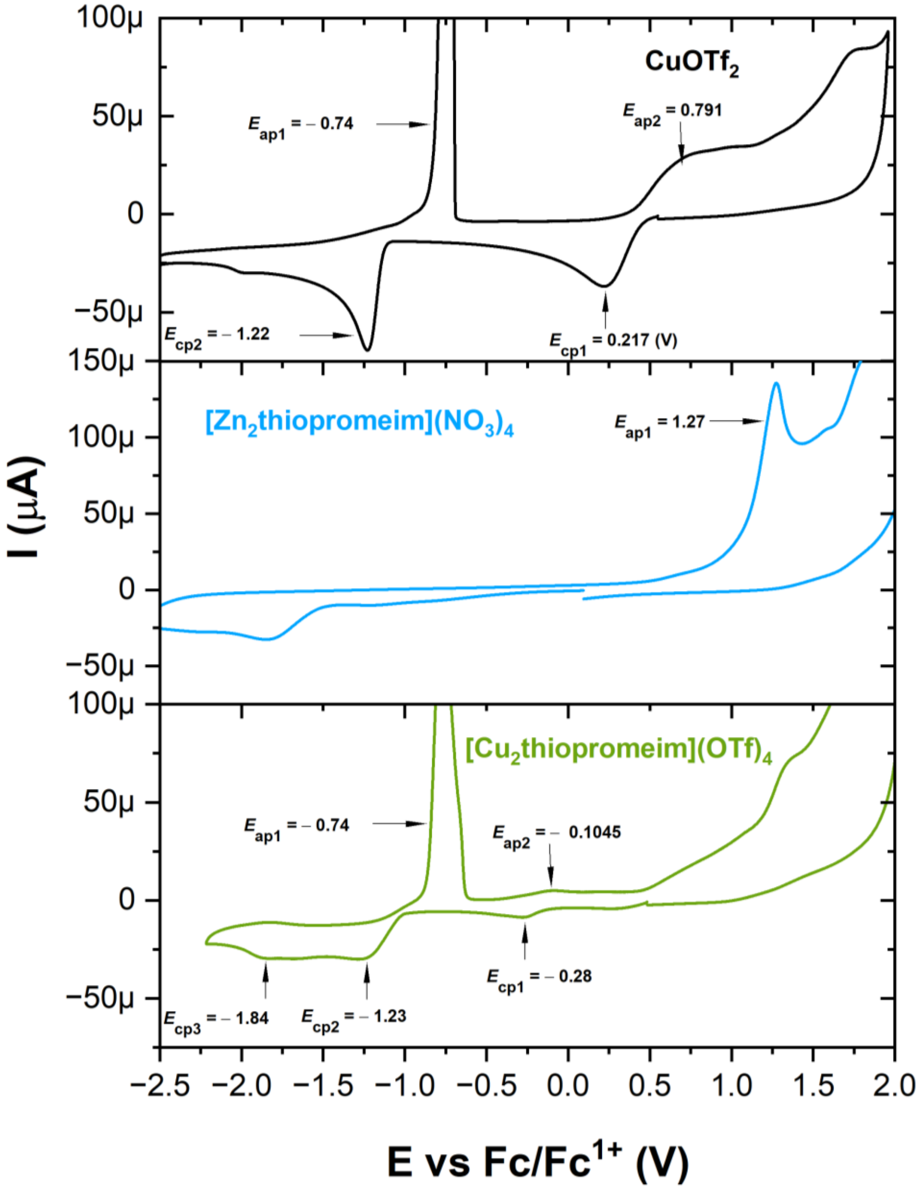

Figure S10. Cathodic comparison voltamperograms of Zn and Cu thiopromeim complexes, 1 mM in CH_3CN at 25°C.

3.8 Superoxide detection by DMPO over time

A

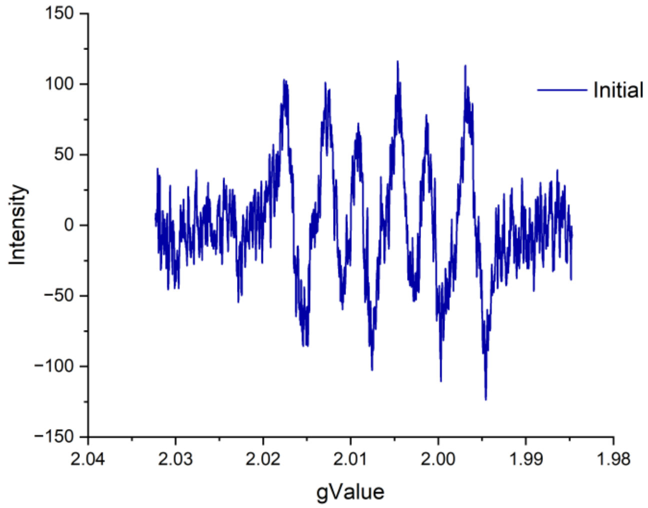

B

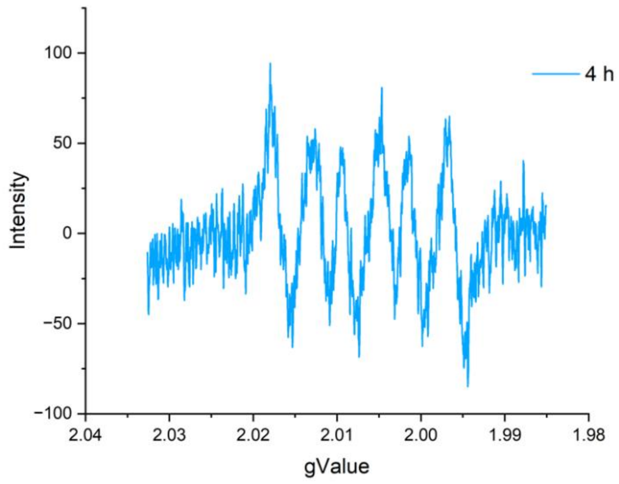

C

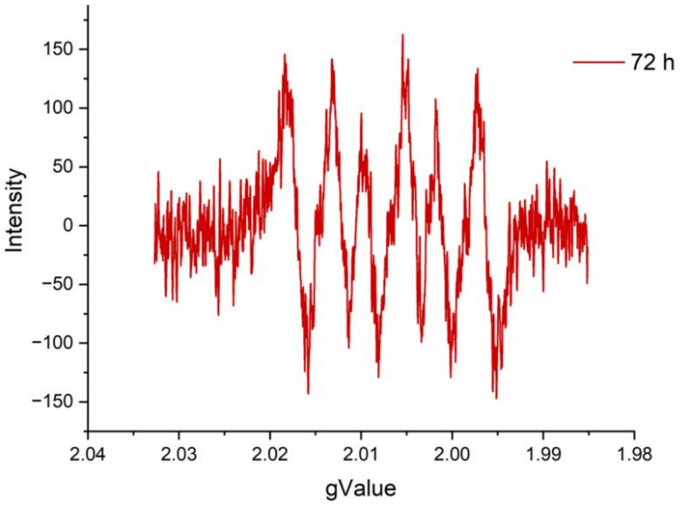

Figure S11. An in-situ prepared solution of [Cu_2*promeim*] 1 mM in MeCN/EtOH 4:1 was kept under an O_2 balloon. Spectra were collected by taking aliquots at A) t = 0, B) t = 4 h and C) 72 h; adding DMPO.

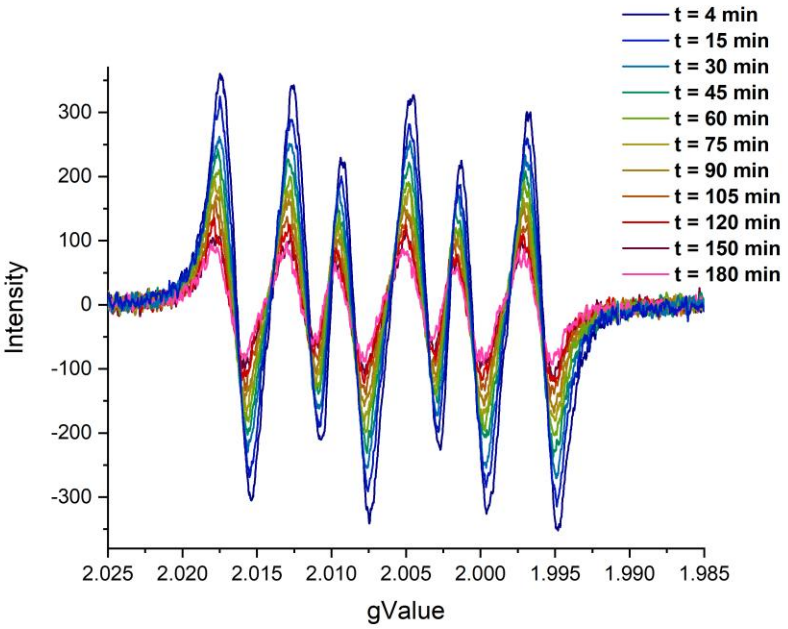

Figure S12. An in-situ prepared solution of [Cu_2*promeim*] 1 mM in MeCN/EtOH 4:1 open. Spectra were collected by taking an aliquot and adding DMPO at different times.

3.9 UV-Vis indirect detection of H_2O_2 over time

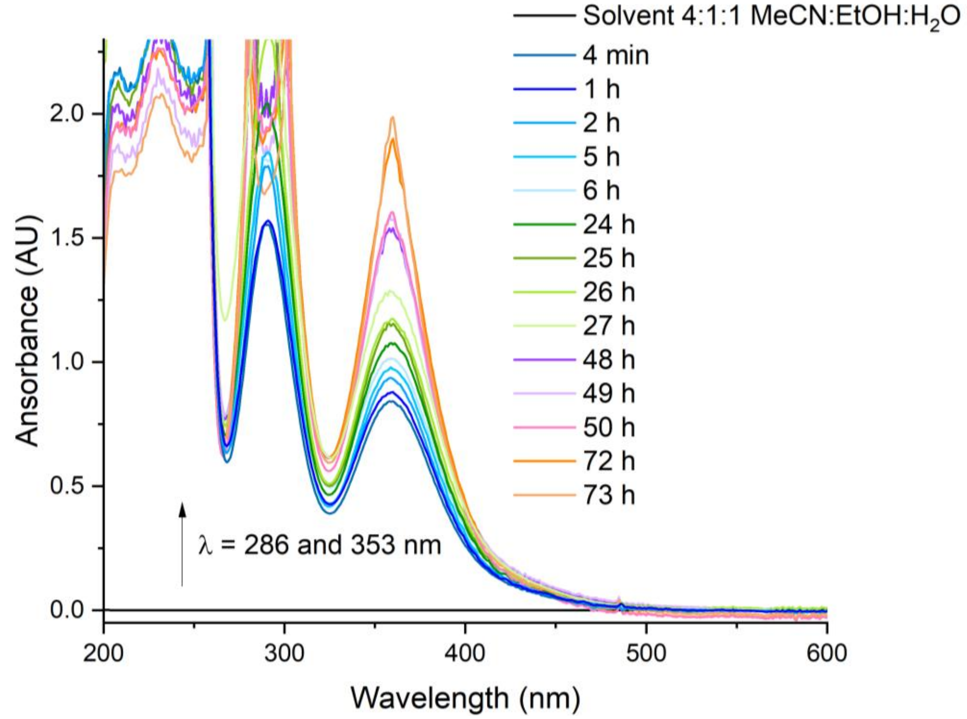

Figure S13. UV-Vis absorption spectra of the transformation of [Cu_2(*promeim*)] + excess $KI_{(ac)}$ at various times. In MeCN, 0.1 mM. I_3^{-1}, λ_{max} = 353 nm, ε^{353} = 26000 M^{-1} cm^{-1}.

3.10 Infrared ATR

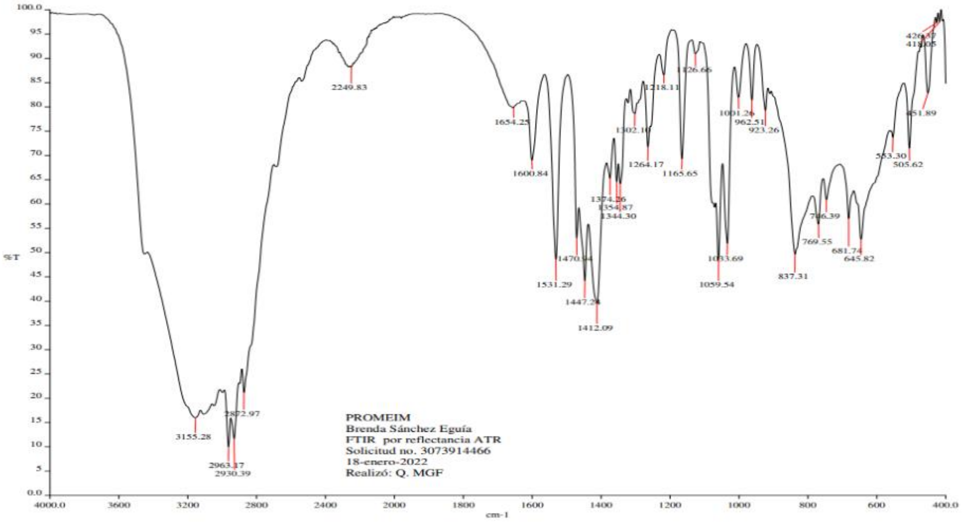

Figure S14. IR-ATR spectrum of *promeim*.

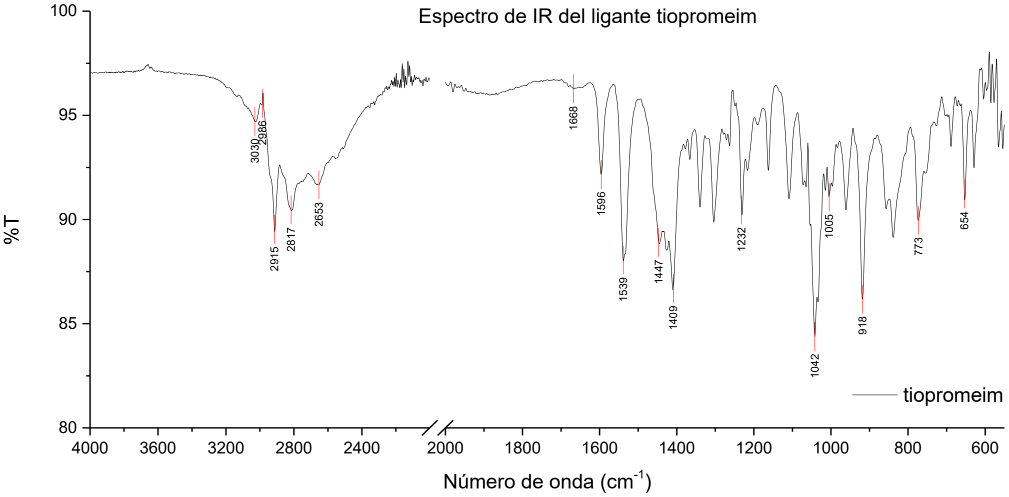

Figure S15. IR-ATR spectrum of *thiopromeim*.

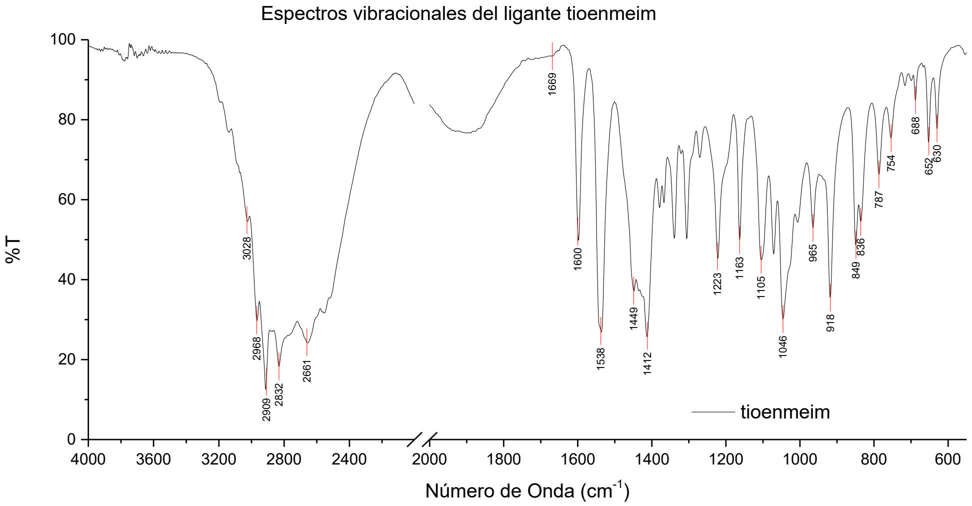

Figure S16. IR-ATR spectrum of *thioenmeim*.

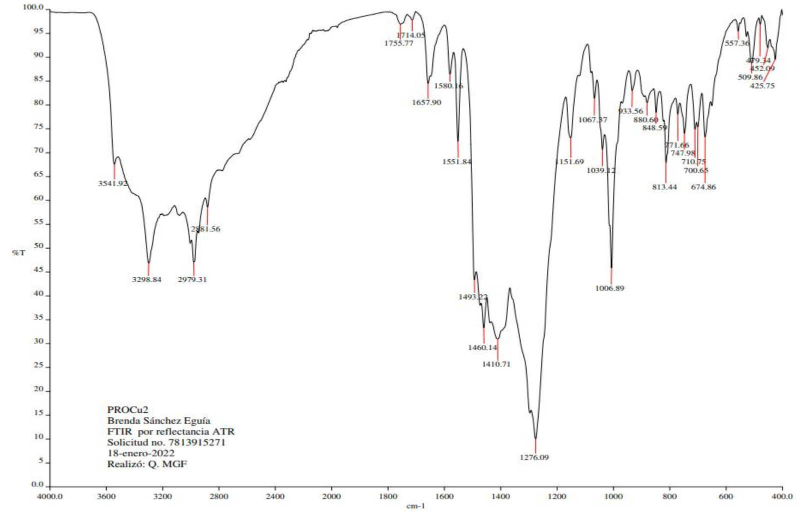

Figure S17. IR-ATR spectrum of [Cu_2*promeim*]$(NO_3)_4$.

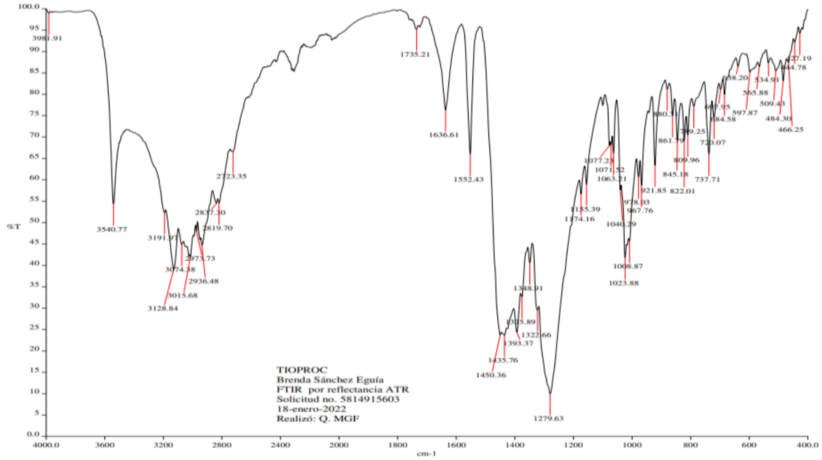

Figure S18. IR-ATR spectrum of [Cu_2*thiopromeim*]$(NO_3)_4$.

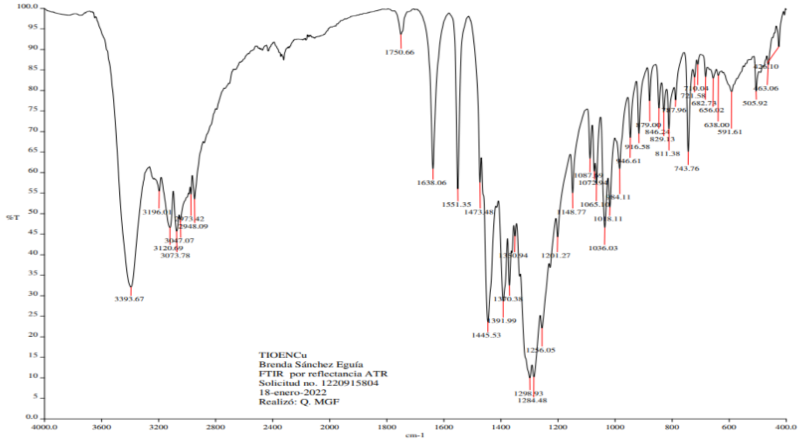

Figure S19. IR-ATR spectrum of [Cu_2*thioenmeim*]$(NO_3)_4$.

3.11 Mass spectrometry

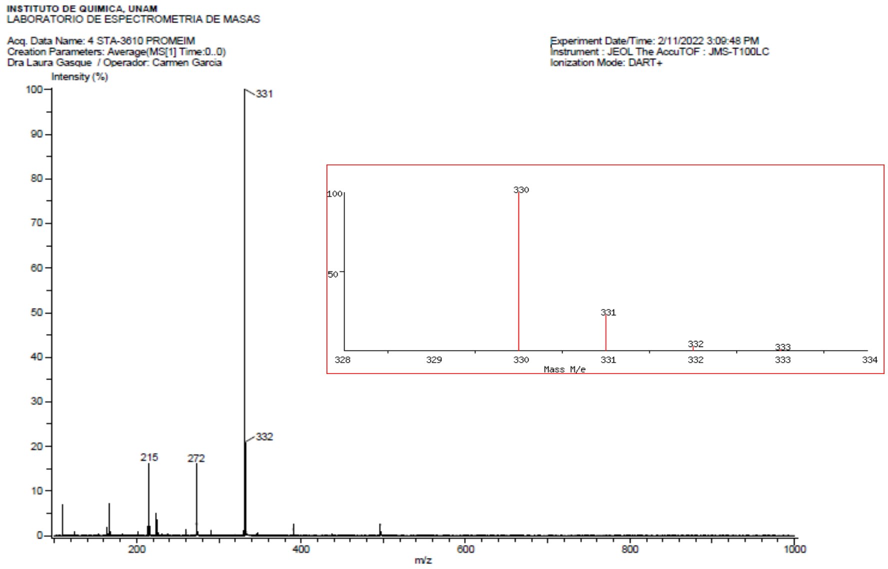

Figure S20. DART-MS spectrum of *promeim* (INSET: Calculated Isotopic distribution).

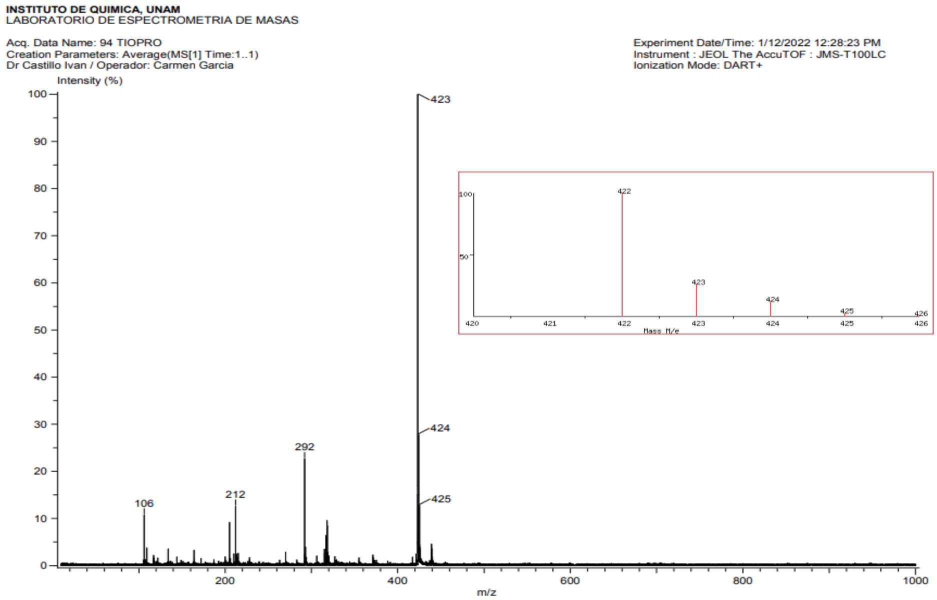

Figure S21. DART-MS spectrum of *thiopromeim* (INSET: Calculated Isotopic distribution).

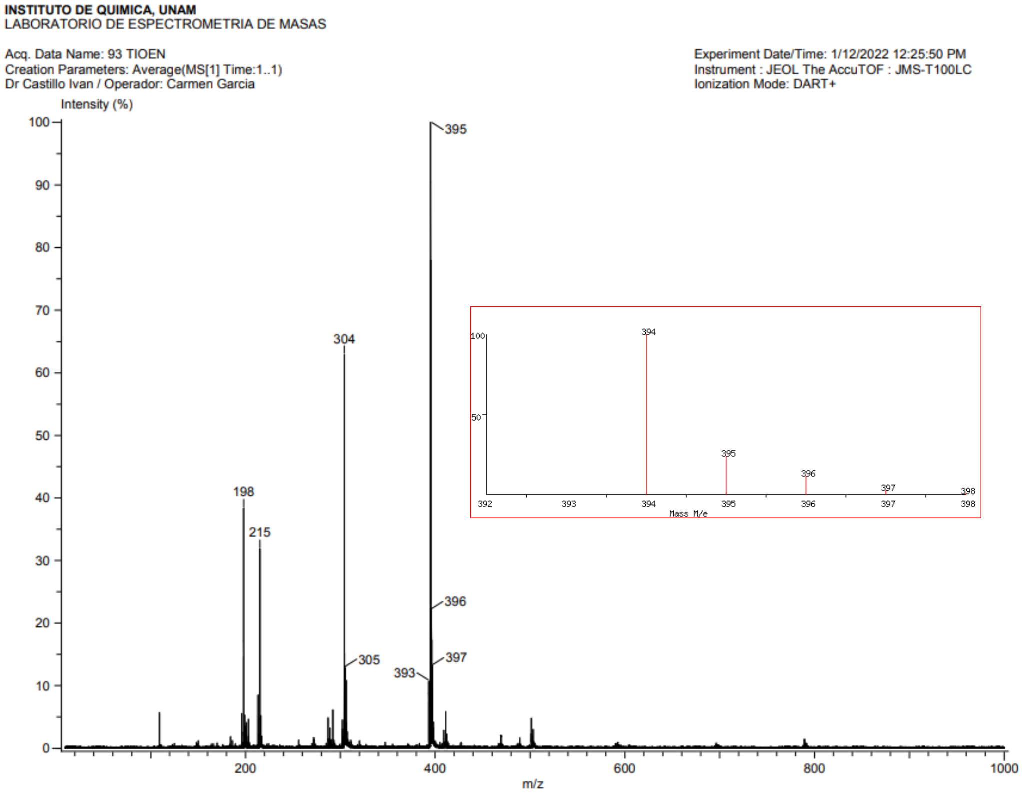

Figure S22. DART-MS spectrum of *thioenmeim* (INSET: Calculated Isotopic distribution).

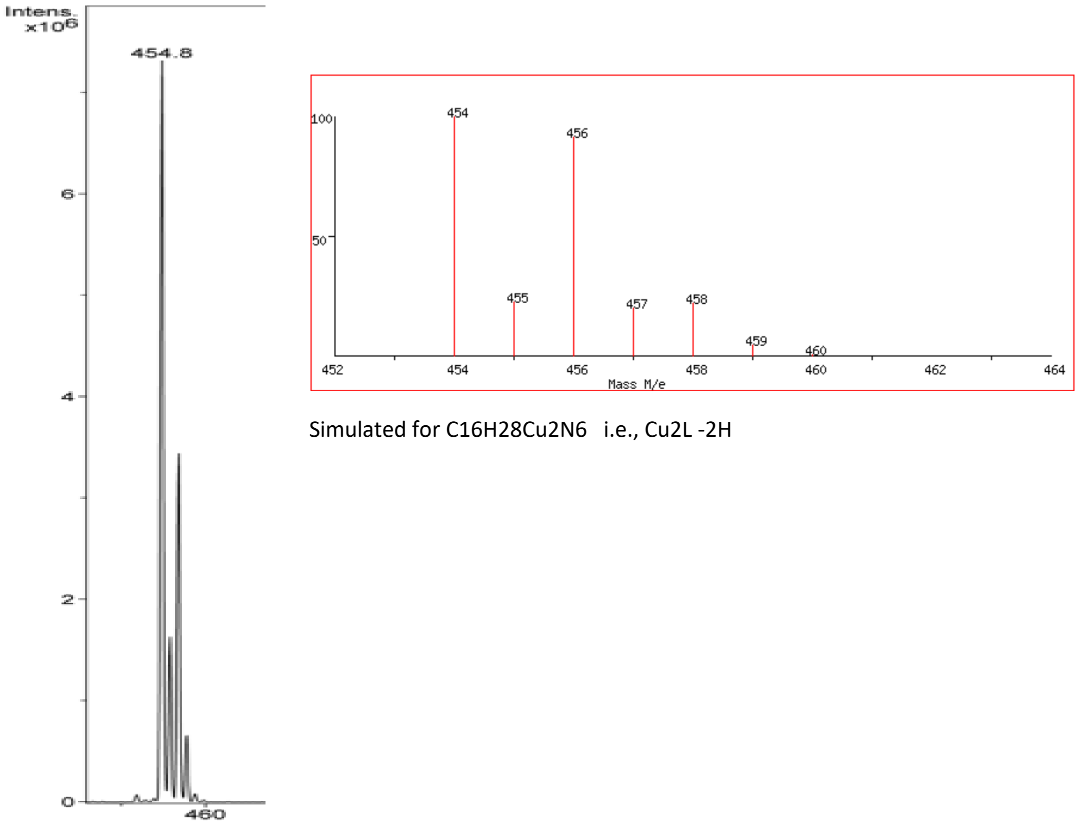

Simulated for C16H28Cu2N6 i.e., Cu2L -2H

Figure S23. ESI-MS spectrum of [Cu_2*promeim*]$(NO_3)_4$ (INSET: Calculated Isotopic distribution).

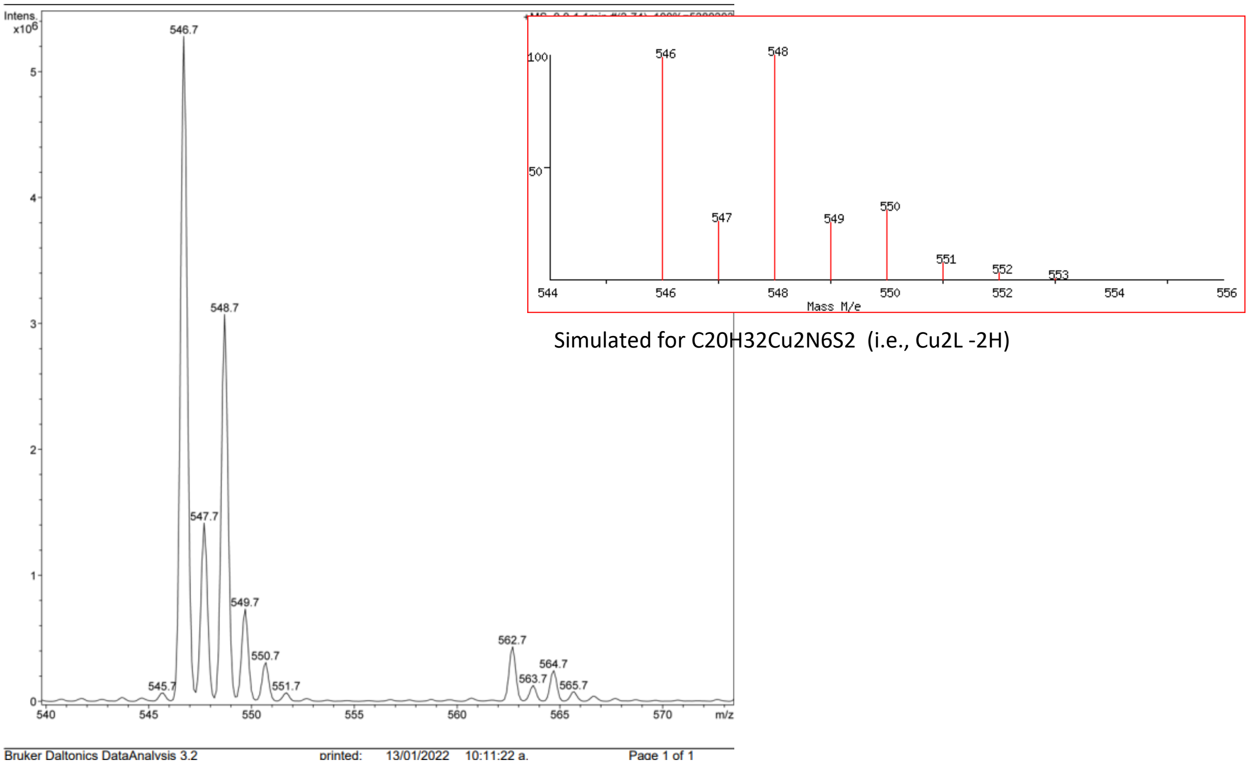

Figure S24. ESI-MS spectrum of [Cu_2*thiopromeim*]$(NO_3)_4$ (INSET: Calculated Isotopic distribution).

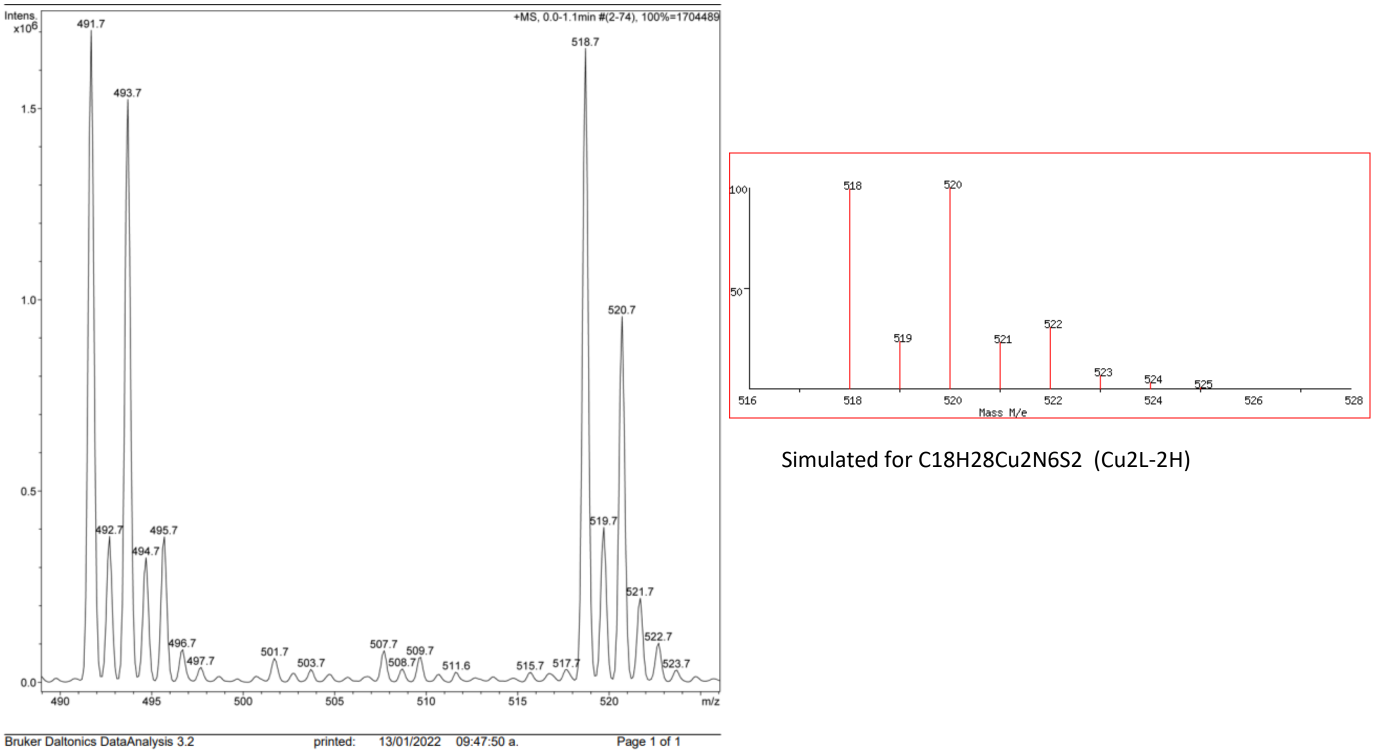

Figure S25. ESI-MS spectrum of [Cu_2*thioenmeim*]$(NO_3)_4$ (INSET: Calculated Isotopic distribution).

3.12 ^{1}H and ^{13}C Nuclear magnetic resonance

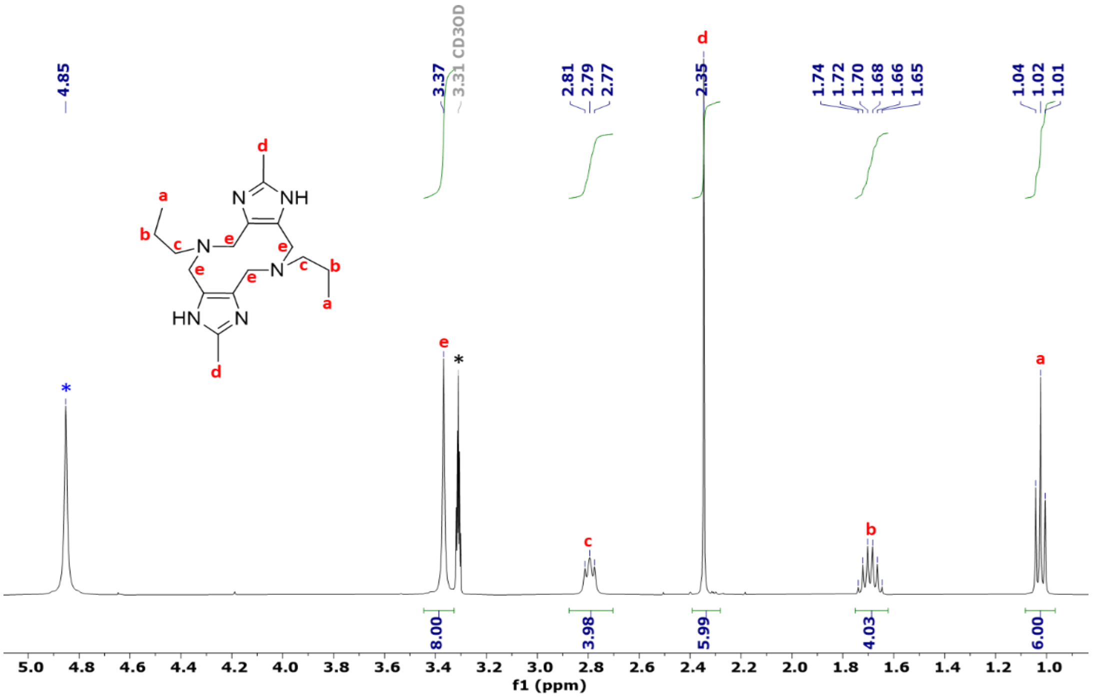

Figure S26. ^{1}H-NMR spectrum of *promeim* in CD_3OD at 25°C. *water, *methanol.

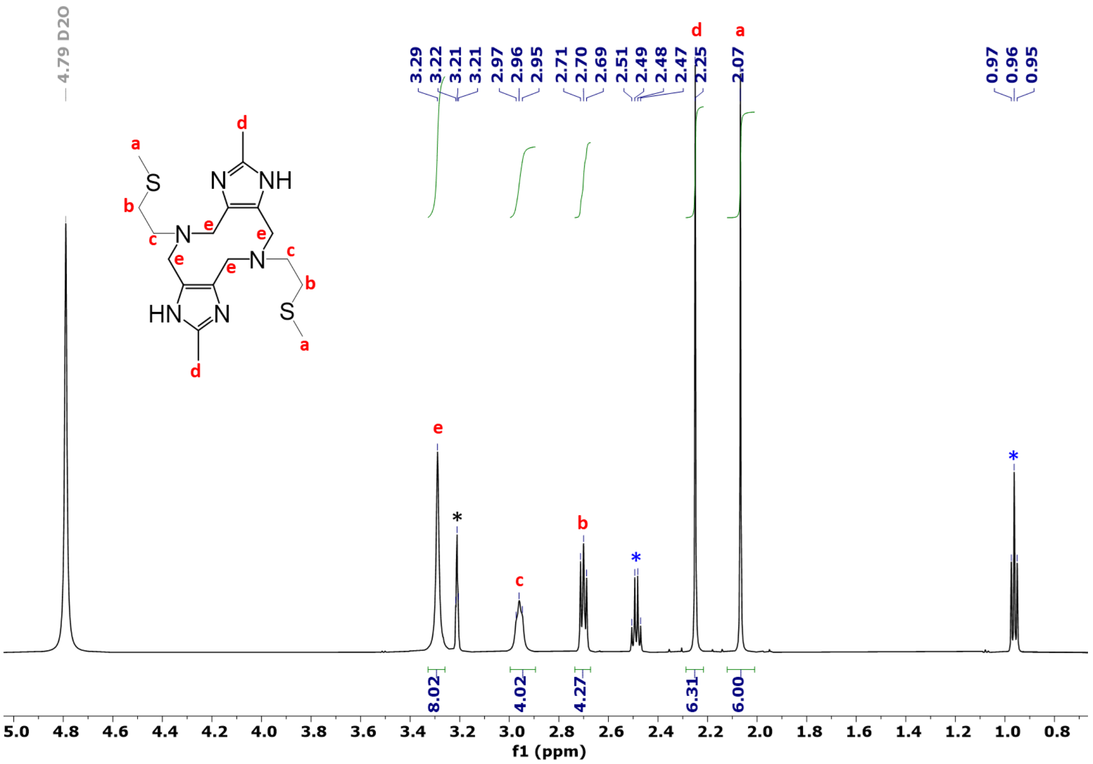

Figure S27. ^{1}H-NMR spectrum of *thioenmeim* in D_2O at 25°C. *ethanol, *methanol.

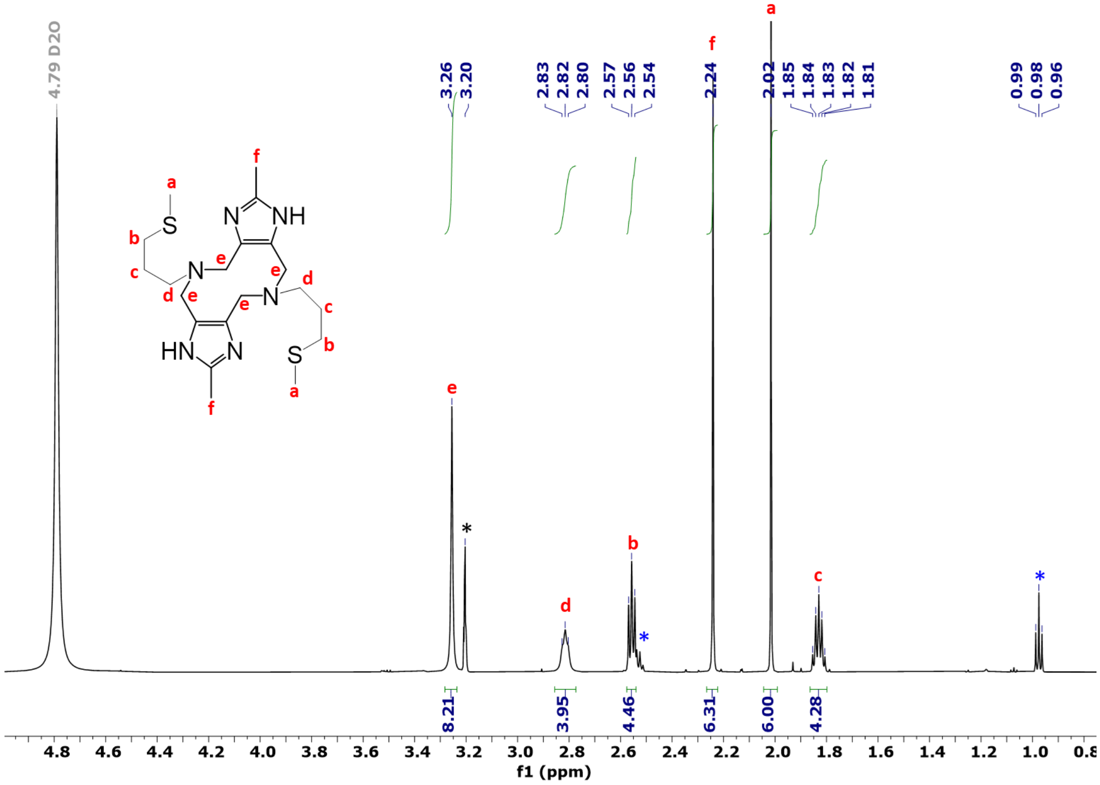

Figure S28. ^{1}H-NMR spectrum of *thiopromeim* in D_2O at 25°C. *ethanol, *methanol.

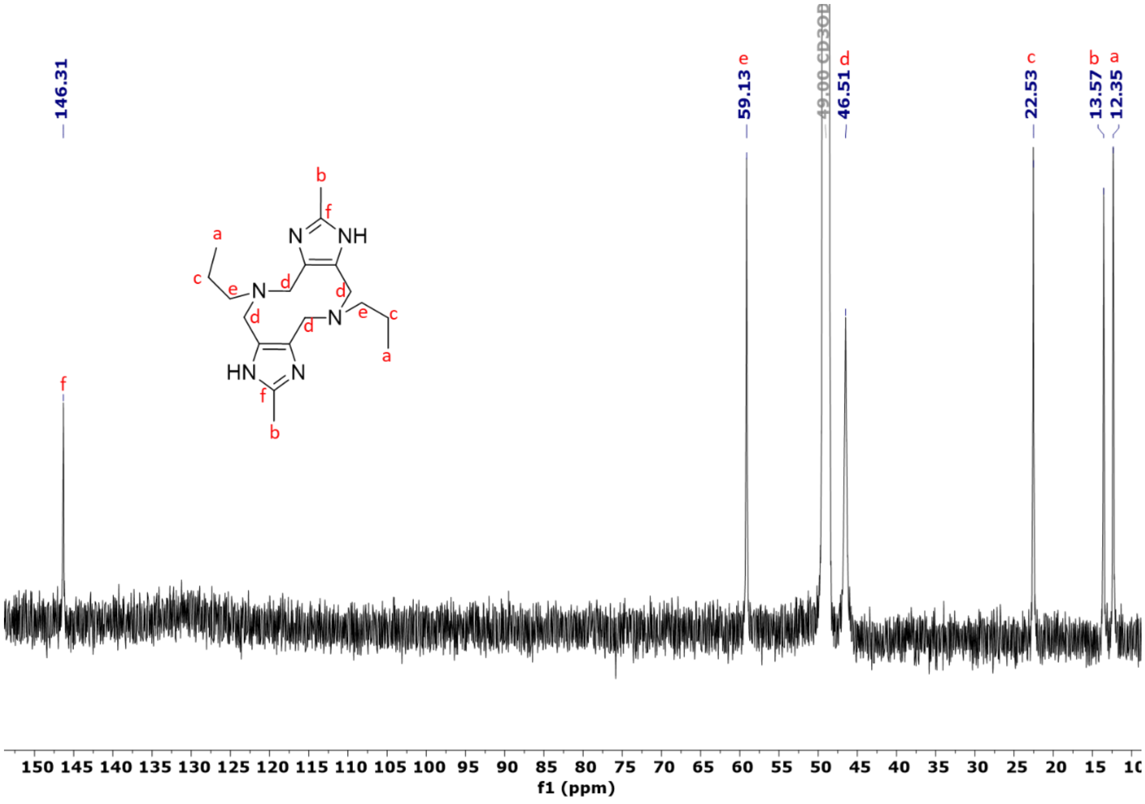

Figure S29. ^{13}C-NMR spectrum of *promeim* in CD_3OD at 25°C.

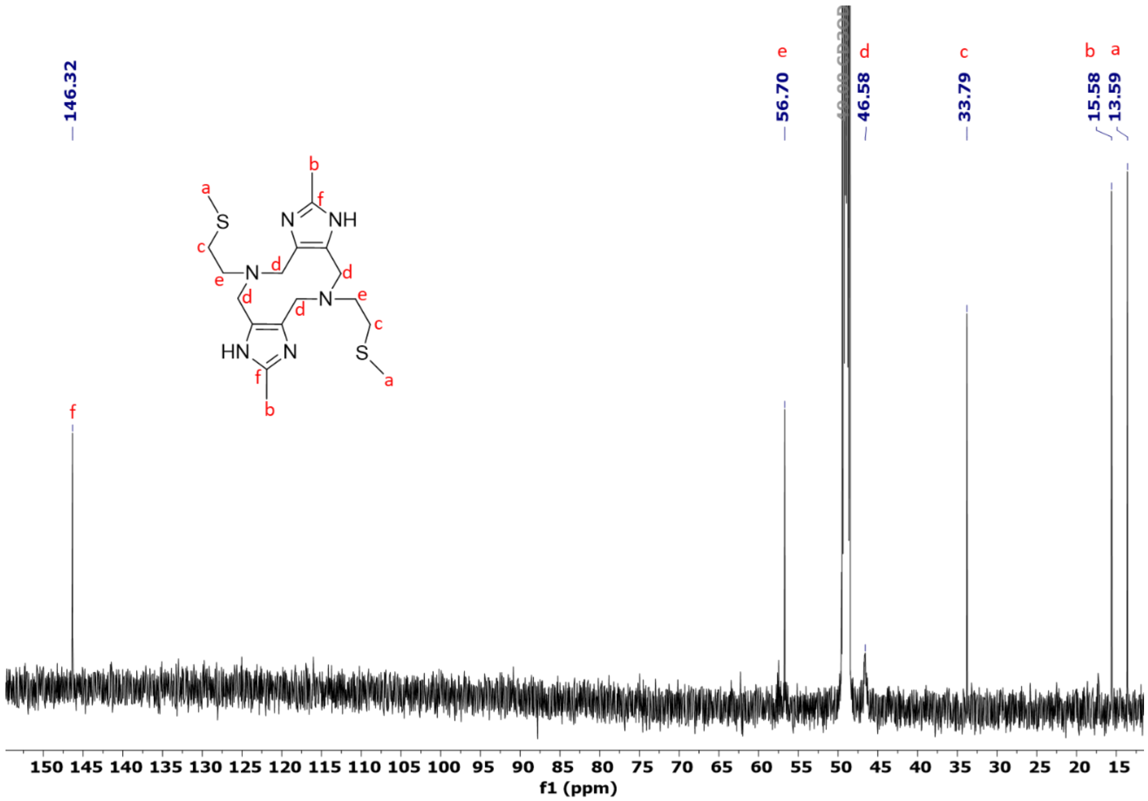

Figure S30. ^{13}C-NMR spectrum of *thioenmeim* in CD_3OD at 25°C.

3.13 DFT calculations

Cartesian coordinates of complexes

Initial complex energy -4938.166619 Hartree/Particle

Cu_2*promeim* initial

C	-1.00969100	-0.17072900	-0.54215000
C	-0.53269000	-0.22710100	0.90003100
H	0.56274000	-0.28756700	0.91874700
H	-0.81558800	0.70416500	1.41495100
C	-2.51079300	-1.41023700	1.73059000
H	-2.93997100	-0.55100100	2.26443600
H	-2.84960000	-1.35469800	0.68991900
C	-2.94125500	-2.71045700	2.29390900
C	-2.80128000	-4.88216800	2.43239300
C	-2.43646700	-6.30019300	2.22813400
H	-1.52282900	-6.35195800	1.62934000
H	-3.23848400	-6.83019100	1.70053900
H	-2.27938900	-6.80460200	3.18766000
C	-0.46242200	-1.26127000	3.12269100
C	-3.87559300	-3.08396100	3.21682200
N	-1.02938100	-1.36796600	1.71949700
N	-2.27236000	-3.82285700	1.82728500
N	-3.77480500	-4.46831900	3.27098500
H	-4.36114800	-5.09070900	3.81911300
C	-4.09675000	-3.33120400	7.81254700

C	-4.62638500	-3.24003800	6.38935900
H	-5.71732100	-3.12835000	6.40100400
H	-4.40316600	-4.19315300	5.88417400
C	-2.60866300	-2.11011300	5.53478300
H	-2.19184400	-2.97909500	5.00623100
H	-2.28429200	-2.18190100	6.57844000
C	-2.17141600	-0.81299100	4.97296500
C	-2.26046100	1.35219700	4.79861600
C	-2.62625000	2.77253600	5.05402200
H	-2.28716800	3.10242500	6.04510700
H	-2.15241500	3.41485800	4.30562800
H	-3.70946100	2.93917800	5.01760400
C	-4.67106900	-2.24157600	4.14651000
H	-4.72591900	-1.23787300	3.71512100
H	-5.70124700	-2.59785800	4.25914400
C	-1.25254400	-0.40062800	4.04068000
N	-4.09617700	-2.13570200	5.54002500
N	-2.82237700	0.29844800	5.45010100
N	-1.30693400	0.97744600	3.94697900
C	-4.30446700	-2.10847100	8.69087900
H	-3.03660900	-3.61647600	7.80608500
H	-4.62075300	-4.18769100	8.25831500
H	-3.78086400	-1.22226700	8.30045700
H	-3.91970600	-2.29041200	9.70045600
H	-5.36794200	-1.85216200	8.77908200
C	-0.67293500	-1.38349600	-1.39363000
H	-0.52790800	0.71759300	-0.97331400
H	-2.08710800	0.03776500	-0.58644800
H	0.40683800	-1.58142700	-1.39697500
H	-0.98782700	-1.22974800	-2.43194600
H	-1.17649900	-2.29546600	-1.03759500
Cu	-4.58380000	-0.15331200	6.16098400
N	-4.80958300	1.54240800	7.25197300
C	-4.88545800	2.50535200	7.88505100
H	-0.41202300	-2.27572000	3.54093500
H	0.56871100	-0.90970200	3.00579700
C	-4.96402800	3.71607500	8.66016700
H	-4.34030400	4.48520500	8.19372300
H	-4.60521900	3.52655600	9.67602400
H	-6.00017400	4.06366800	8.70063000
Cu	-0.42642200	-3.30897300	1.16058900
N	1.50474500	-2.73385800	1.00231100
C	2.63732100	-2.51749700	0.92554000
C	4.04697500	-2.24086500	0.82651400
H	4.55402900	-2.58901200	1.73115600
H	4.20355900	-1.16400900	0.71456000
H	4.46196700	-2.75996300	-0.04237700
N	-6.49725600	-0.64638200	6.49613700
C	-7.62080200	-0.85271800	6.66508500
C	-9.01975800	-1.11824700	6.87671700
H	-9.16059900	-2.17895300	7.10457500
H	-9.58214400	-0.86357200	5.97358100
H	-9.38494000	-0.51577800	7.71358300
O	0.06380600	-5.08919400	0.75628800
H	0.88652700	-5.07522100	0.25164800
O	0.86076600	-5.45350200	3.39143000

H	0.59086700	-5.39834100	2.44398200
H	1.49374800	-4.73599400	3.50887300
O	-5.61061600	0.85967900	4.16589000
H	-5.14945400	1.59578600	3.74091500
H	-6.51880400	1.16465300	4.29663900

Calculation of the energy difference between the protonated species HRef (initial complex) and the deprotonated species Ref⁻ (final complex).

For pKa calculation, the promeim complex was the reactant and the product was the deprotonated one.

$$[Cu_2L(CH_3CN)_3(H_2O)_2OH] \longrightarrow [Cu_2L\text{-}H(CH_3CN)_3(H_2O)_2OH]$$

The calculation was performed using a reference method,

$$\mathrm{HA} + \mathrm{Ref}^{-} \leftrightarrow \mathrm{A}^{-} + \mathrm{HRef}$$

For our case, the reference compound was the imidazole and deprotonated imidazole, according to the following expression.

$$\mathrm{p}K_\mathrm{a} = \frac{\Delta G^0}{RT\ln(10)} + \mathrm{p}K_\mathrm{a}(\mathrm{HRef})$$

According to literature[11] the taken pKa value of imidazole was 15.05, the standard-state ΔG from pH = 0 to pH = 7 was corrected by the factor 7.0 × 1.36, and this value is subtracted from the change in G.

Deprotonated complex energy -4937.716580 Hartree/Particle

Cu_2*promeim-H*

C	0	-1.0096913478	-0.1707287153	-0.54214997
C	0	-0.5326901083	-0.2271014037	0.9000312713
H	0	0.5627397594	-0.2875668058	0.9187465989
H	0	-0.8155880894	0.7041650386	1.4149507343
C	0	-2.510793498	-1.4102370512	1.7305899205
H	0	-2.9399714031	-0.5510007838	2.2644363735
H	0	-2.8495997411	-1.3546978977	0.6899193996
C	0	-2.9412552829	-2.7104569206	2.2939087209
C	0	-2.8012796889	-4.8821675908	2.4323934387
C	0	-2.4364672113	-6.3001925913	2.2281344432
H	0	-1.5228290012	-6.3519580821	1.6293398356
H	0	-3.2384838614	-6.8301913413	1.7005390968
H	0	-2.279389499	-6.8046019177	3.1876595429
C	0	-0.462422283	-1.2612698253	3.1226908288
C	0	-3.8755934564	-3.0839614927	3.2168222323
N	0	-1.0293808937	-1.3679661318	1.7194970207
N	0	-2.272360467	-3.8228572289	1.8272852177
N	0	-3.7748053288	-4.4683191104	3.270985379
H	0	-4.36114847	-5.0907094273	3.8191132377
C	0	-4.0967498928	-3.3312041579	7.8125471937
C	0	-4.6263851719	-3.240037975	6.3893593031
H	0	-5.7173209491	-3.1283502453	6.4010035486
H	0	-4.4031657076	-4.1931526188	5.884174492
C	0	-2.60866269	-2.1101129957	5.5347829375
H	0	-2.1918436881	-2.9790952773	5.0062308241
H	0	-2.2842924946	-2.1819009289	6.5784395813
C	0	-2.1714163882	-0.8129912729	4.9729648717

C	0	-2.2604606087	1.3521968669	4.7986164876
C	0	-2.6262499664	2.7725357302	5.0540221914
H	0	-2.2871683723	3.1024250723	6.0451072623
H	0	-2.1524154143	3.4148581	4.3056276628
H	0	-3.7094608408	2.9391777046	5.0176041024
C	0	-4.6710693728	-2.2415759434	4.1465100046
H	0	-4.7259189793	-1.2378732173	3.7151214904
H	0	-5.7012473076	-2.5978583225	4.2591435204
C	0	-1.2525435451	-0.4006282273	4.0406796251
N	0	-4.0961774881	-2.1357016444	5.5400251038
N	0	-2.8223765395	0.2984482579	5.4501013154
N	0	-1.3069338368	0.977445664	3.9469794882
C	0	-4.3044665738	-2.1084709385	8.6908791895
H	0	-3.0366087081	-3.6164758946	7.8060845749
H	0	-4.6207527815	-4.1876910496	8.2583148127
H	0	-3.7808640918	-1.2222666178	8.3004567519
H	0	-3.9197063599	-2.2904117375	9.7004561037
H	0	-5.3679415567	-1.8521615229	8.7790815986
C	0	-0.6729353782	-1.3834959689	-1.3936298612
H	0	-0.5279084587	0.7175931594	-0.9733138295
H	0	-2.0871081971	0.0377651854	-0.5864478979
H	0	0.4068379071	-1.581427009	-1.3969754076
H	0	-0.9878265074	-1.2297479611	-2.4319463226
H	0	-1.176498867	-2.2954660958	-1.0375950113
Cu	0	-4.5838004906	-0.1533124703	6.1609843299
N	0	-4.8095832029	1.5424078039	7.2519731483
C	0	-4.885458189	2.5053523534	7.8850510223
H	0	-0.4120234161	-2.2757196083	3.5409346832
H	0	0.5687108746	-0.9097016126	3.0057974386
C	0	-4.96402765	3.716074555	8.6601672519
H	0	-4.3403038709	4.4852049462	8.1937229618
H	0	-4.6052185397	3.5265560438	9.6760240373
H	0	-6.0001735628	4.0636677086	8.7006299909
Cu	0	-0.4264222507	-3.3089729469	1.1605890279
N	0	1.504744829	-2.7338576006	1.0023108747
C	0	2.637320763	-2.5174968973	0.9255404791
C	0	4.0469749087	-2.2408649091	0.8265144025
H	0	4.554028904	-2.5890123656	1.7311555601
H	0	4.203558688	-1.1640085607	0.7145595414
H	0	4.4619670382	-2.7599625531	-0.042376524
N	0	-6.4972563341	-0.6463824501	6.4961369885
C	0	-7.6208023365	-0.8527176965	6.6650850278
C	0	-9.0197575471	-1.1182465278	6.8767174029
H	0	-9.1605988	-2.1789530198	7.1045749767
H	0	-9.5821441932	-0.8635723852	5.9735810056
H	0	-9.3849401635	-0.5157775183	7.7135833641
O	0	0.0638063706	-5.0891936198	0.7562881289
H	0	0.8865271515	-5.0752209246	0.2516477677
O	0	0.8607660304	-5.4535017978	3.3914298241
H	0	0.5908666631	-5.3983411558	2.443981969
H	0	1.4937484684	-4.7359943121	3.5088733144
O	0	-5.610616379	0.8596787653	4.1658900293
H	0	-5.149454113	1.5957856124	3.7409153016

H	0	-6.5188039499	1.1646533044	4.2966386438

Final complex energy -3088.471516 Hartree/Particle

Cu_2*promeim* final

C	-1	-1.42358800	0.83897000	0.84296600
H	-1	-2.32215200	0.34944700	0.45290900
H	-1	-1.65509900	1.90675900	0.94598800
C	-1	-0.26758500	0.62949200	-0.10828700
H	-1	0.63199100	1.12541600	0.29007200
H	-1	-0.04096600	-0.44337100	-0.16429400
C	-1	0.78589400	0.76225900	-2.25634100
H	-1	1.62336900	1.38920000	-1.92448700
H	-1	1.04167900	-0.27470100	-2.00883400
C	-1	0.52312000	0.87300200	-3.70465900
C	-1	-0.70847900	0.48635100	-5.45482200
C	-1	-1.75894700	-0.00030700	-6.37071400
H	-1	-2.70925200	-0.08246800	-5.83756400
H	-1	-1.50398400	-0.99145600	-6.76197900
H	-1	-1.87726500	0.67774900	-7.22029600
C	-1	-0.85202300	2.50777600	-1.65170700
H	-1	-1.32170600	2.64230600	-2.63080700
H	-1	-1.61410000	2.72675300	-0.90001700
C	-1	1.11648300	1.54681800	-4.72645900
N	-1	-0.46620200	1.07151200	-1.51400400
N	-1	-0.60587500	0.22893200	-4.16066100
N	-1	0.31929400	1.27707500	-5.82769200
H	-1	0.48828200	1.59428400	-6.77744200
C	-1	2.98576900	4.33737500	-7.00614200
H	-1	3.82196100	4.66972800	-6.37373400
H	-1	3.19466100	3.29580900	-7.28150300
C	-1	1.70057900	4.43424800	-6.20554200
H	-1	0.87619500	3.94207000	-6.75862700
H	-1	1.42149200	5.49522100	-6.12539800
C	-1	0.64378600	4.27141600	-4.04062100
H	-1	-0.24095200	3.66504100	-4.29159300
H	-1	0.38374500	5.31922500	-4.25057000
C	-1	0.94653000	4.18113800	-2.58999100
C	-1	1.96263300	4.49830500	-0.72703200
C	-1	2.97262200	5.01426700	0.20125200
H	-1	3.00544800	6.10928800	0.14943900
H	-1	2.76778600	4.69781300	1.22406000
H	-1	3.96813600	4.66236800	-0.09934900
C	-1	2.23021600	2.53616400	-4.69192100
H	-1	2.72735600	2.41373500	-3.72057100
H	-1	2.98535200	2.31934100	-5.45189900
C	-1	0.28571900	3.45526000	-1.51200200
N	-1	1.81976800	3.94051800	-4.83475500
N	-1	1.99011600	4.80466900	-2.06486800
N	-1	0.93776700	3.68656100	-0.37828000
Cu	0	3.27172500	5.66668500	-3.23424800
N	0	4.10835700	6.81719800	-4.48311100
C	0	4.57866800	7.36934200	-5.37527300
C	0	5.16353000	8.04439400	-6.49825200
H	0	4.44186100	8.74435500	-6.92661500

H	0	6.05234500	8.59426300	-6.17890400
H	0	5.44843000	7.31039400	-7.25703900
C	-1	2.90530700	5.18521700	-8.26222900
H	-1	3.81307900	5.09681400	-8.86680600
H	-1	2.05883700	4.88219500	-8.89020400
H	-1	2.76762400	6.24494200	-8.01463600
C	-1	-1.07326400	0.26823000	2.20596900
H	-1	-0.17955200	0.74987100	2.61965900
H	-1	-1.89001300	0.41378400	2.91881200
H	-1	-0.87213700	-0.80772000	2.14396100
Cu	-1	-1.90030200	-0.05325800	-2.64422300
N	-1	-2.98001900	2.71126500	-4.54715300
C	-1	-2.63870400	3.19940400	-5.53490400
C	-1	-2.19605400	3.80216800	-6.76730300
H	-1	-1.52557300	4.63931400	-6.55296200
H	-1	-3.05433300	4.17090200	-7.33460700
H	-1	-1.66340100	3.06083600	-7.37050100
N	-1	-3.54732000	0.62092300	-1.67459500
C	-1	-4.58604400	0.95161700	-1.31499000
C	-1	-5.88273600	1.36304100	-0.85872400
H	-1	-6.46221800	0.48484700	-0.56380500
H	-1	-6.40568000	1.88560700	-1.66329700
H	-1	-5.77896700	2.03193900	-0.00119200
O	-1	-2.92195800	-1.45378600	-3.38063600
H	-1	-2.42862700	-1.87062900	-4.09345200
O	0	5.08845200	4.14816900	-3.15967600
H	0	4.89362800	3.24562100	-2.88177600
H	0	5.49468800	4.06247800	-4.03027400
O	-1	-3.13300200	-2.67650200	-0.93695000
H	-1	-3.09001200	-2.32852600	-1.85557100
H	-1	-2.63321800	-3.49626800	-0.95598500

4 References

1. CrysAlisPro, version 1.171.36.32; Oxford Diffraction Ltd.: Abingdon, U.K., 2013.
2. Clark, R. C.; Reid, J. S. The analytical calculation of absorption in multifaceted crystals. Acta Cryst. 1995, A51, 887-897. doi.org/10.1107/S0108767395007367
3. Sheldrick, G. M. SHELXT - Integrated space-group and crystal-structure determination; Acta Cryst. 2015, A71, 3-8 doi.org/10.1107/S2053273314026370
4. Sheldrick, G. M. Crystal structure refinement with SHELXL; Acta Cryst. 2015, C71, 3-8 doi.org/10.1107/S2053229614024218
5. Farrugia L.J. WinGX and ORTEP for Windows: an update. J. Appl. Cryst. 2012, 45, 849-854 doi.org/10.1107/S0021889812029111
6. Macrae, C.F.; Edgington, P.R.; McCabe, P.; Pidcock, E.; Shields, G.P.; Taylor, R.; Towler, M.; and van de Streek, J. Mercury: visualisation and analysis of crystal structures. J. Appl. Crystallogr. 2006 39, 453–457. doi:10.1107/S002188980600731X
7. Frisch, M. J.; Trucks, G. W.; Schlegel, H. B.; Scuseria, G. E.; Robb, M. A.; Cheeseman, J. R.; Scalmani, G.; Barone, V.; Mennucci, B.; Petersson, G. A.; Nakatsuji, H.; Caricato, M.; Li, X.; Hratchian, H. P.; Izmaylov, A. F.; Bloino, J.; Zheng, G.; Sonnenberg, J. L.; Hada, M.; Ehara, M.; Toyota, K.; Fukuda, R.; Hasegawa, J.; Ishida, M.; Nakajima, T.; Honda, Y.; Kitao, O.; Nakai, H.; Vreven, T.; Montgomery Jr., J. A.; Peralta, J. E.; Ogliaro, F.; Bearpark, M. J.; Heyd, J.; Brothers, E. N.; Kudin, K. N.; Staroverov, V. N.; Kobayashi, R.; Normand, J.; Raghavachari, K.; Rendell, A. P.; Burant, J. C.; Iyengar, S. S.; Tomasi, J.; Cossi, M.; Rega, N.; Millam, N. J.; Klene, M.; Knox, J. E.; Cross, J. B.; Bakken, V.; Adamo, C.; Jaramillo, J.; Gomperts, R.; Stratmann, R. E.; Yazyev, O.; Austin, A. J.; Cammi, R.; Pomelli, C.; Ochterski, J. W.; Martin, R. L.; Morokuma, K.; Zakrzewski, V. G.; Voth, G. A.; Salvador, P.; Dannenberg, J. J.; Dapprich, S.; Daniels, A. D.; Farkas, Ö.; Foresman, J. B.; Ortiz, J. V.; Cioslowski, J.; Fox, D. J.; Gaussian, Inc.: Wallingford, CT, USA, 2009.

8. Marenich, A. V.; Cramer, C. J.; Truhlar, D. G. J Phys Chem B 2009, 113, 6378-6396.
9. Zhao, Y.; Truhlar, D. G. Theor Chem Acc 2008, 120, 215-241.
10. Zhao, Y.; Schultz, N. E.; Truhlar, D. G. J Chem Theory Comput 2006, 2, 364-382.
11. Lõkov, M.; Tshepelevitsh, S.; Heering, A.; Plieger, P. G.; Vianello, R.; Leito, I. Eur. J. Org. Chem. 2017, 4475–4489.
